# Supplementary material for: Population heterogeneity in Plasmodium vivax relapse risk
Source: PLoS Negl Trop Dis. 2022 Dec 19;16(12):e0010990. doi: 10.1371/journal.pntd.0010990 (PMC9810152; doi:10.1371/journal.pntd.0010990)
Supplement: S1 Figs — (PDF) [file pntd.0010990.s001.pdf]

S2 Figures

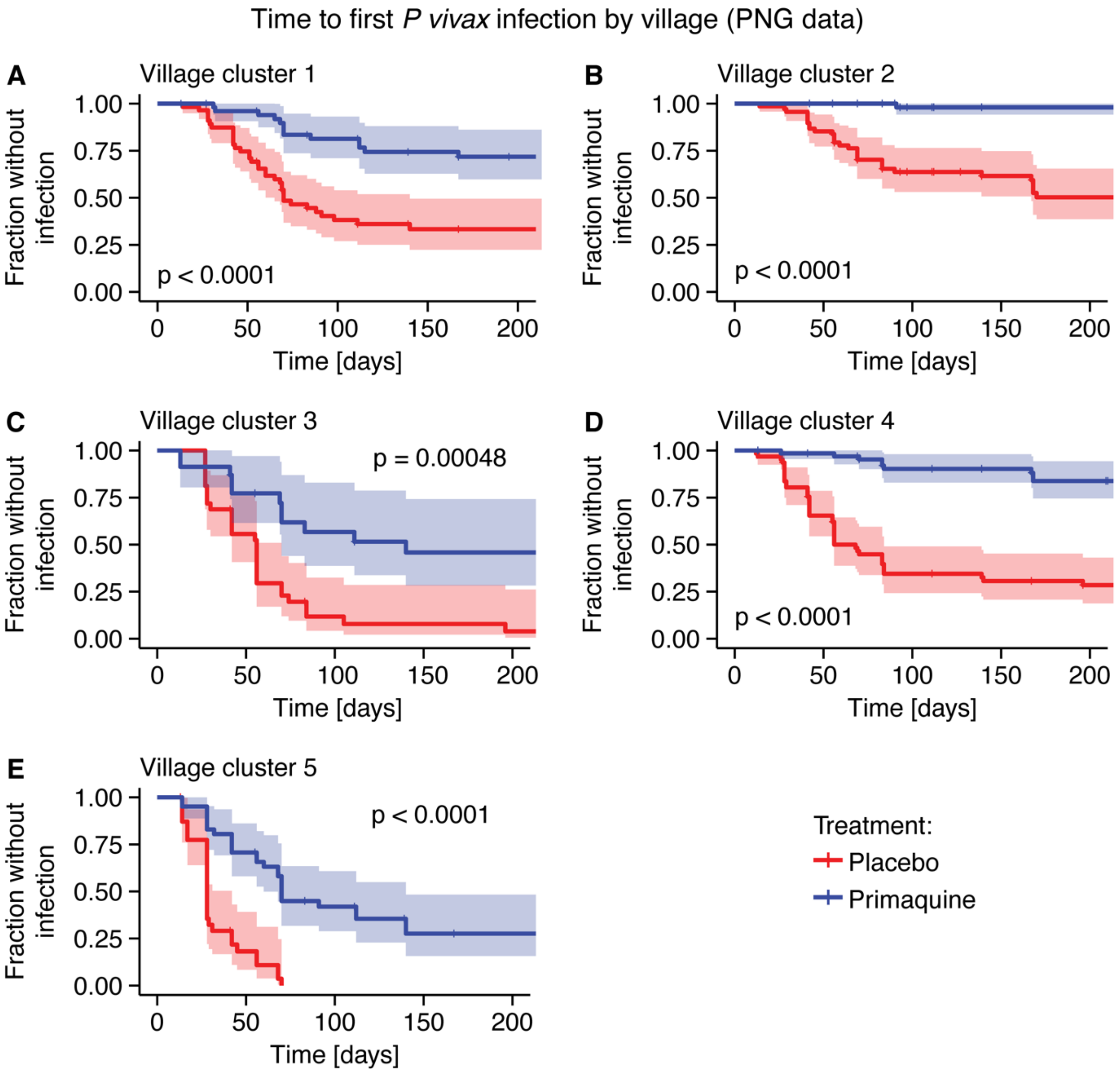

Fig A Time to first P vivax infection by village in the PNG data for patients treated with primaquine (blue) and a placebo (red). The placebo-treated patients have a higher infection risk in all villages (log-rank test with p-values < 0.001, see figures). Fits of the constant relapse risk, temporal heterogeneity, population heterogeneity, and temporal and population heterogeneity models all show different risks of new, mosquito-borne infections between villages with the lowest risk in village 2, followed by villages 4, 1, 3, and the highest risk in village 5 (see **Table E**, **Table F**, **Table G**, and **Table H** in **S1 Tables**).

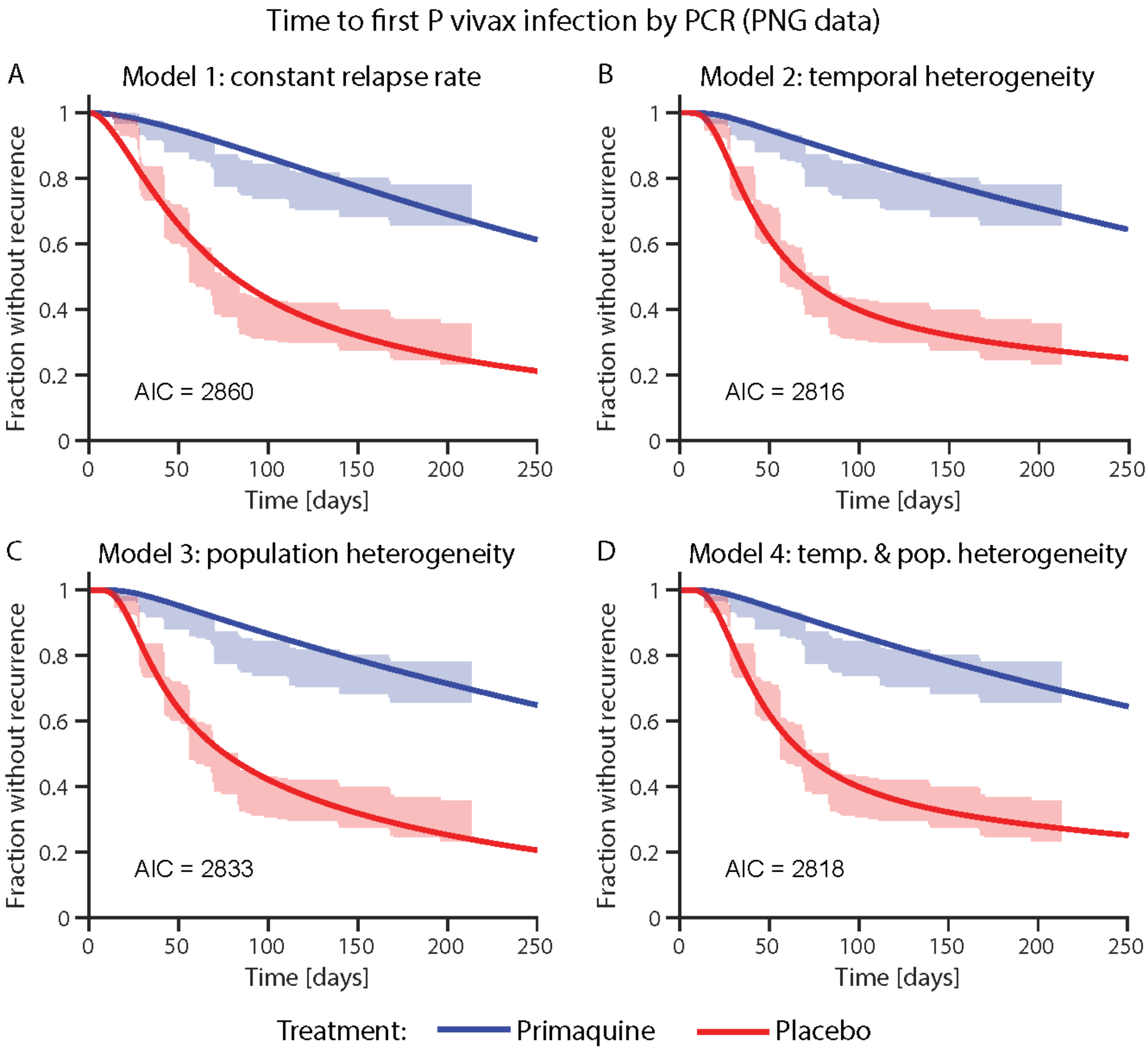

Fig B Model fit of the constant relapse rate, temporal heterogeneity, the population heterogeneity, and temporal and population heterogeneity model to the first recurrence time in the PNG data. The shaded areas are the 95% confidence regions from the data. For the parameters of the model fit see **Tables A**, **B**, **C** and **D** in **S1 Tables**.

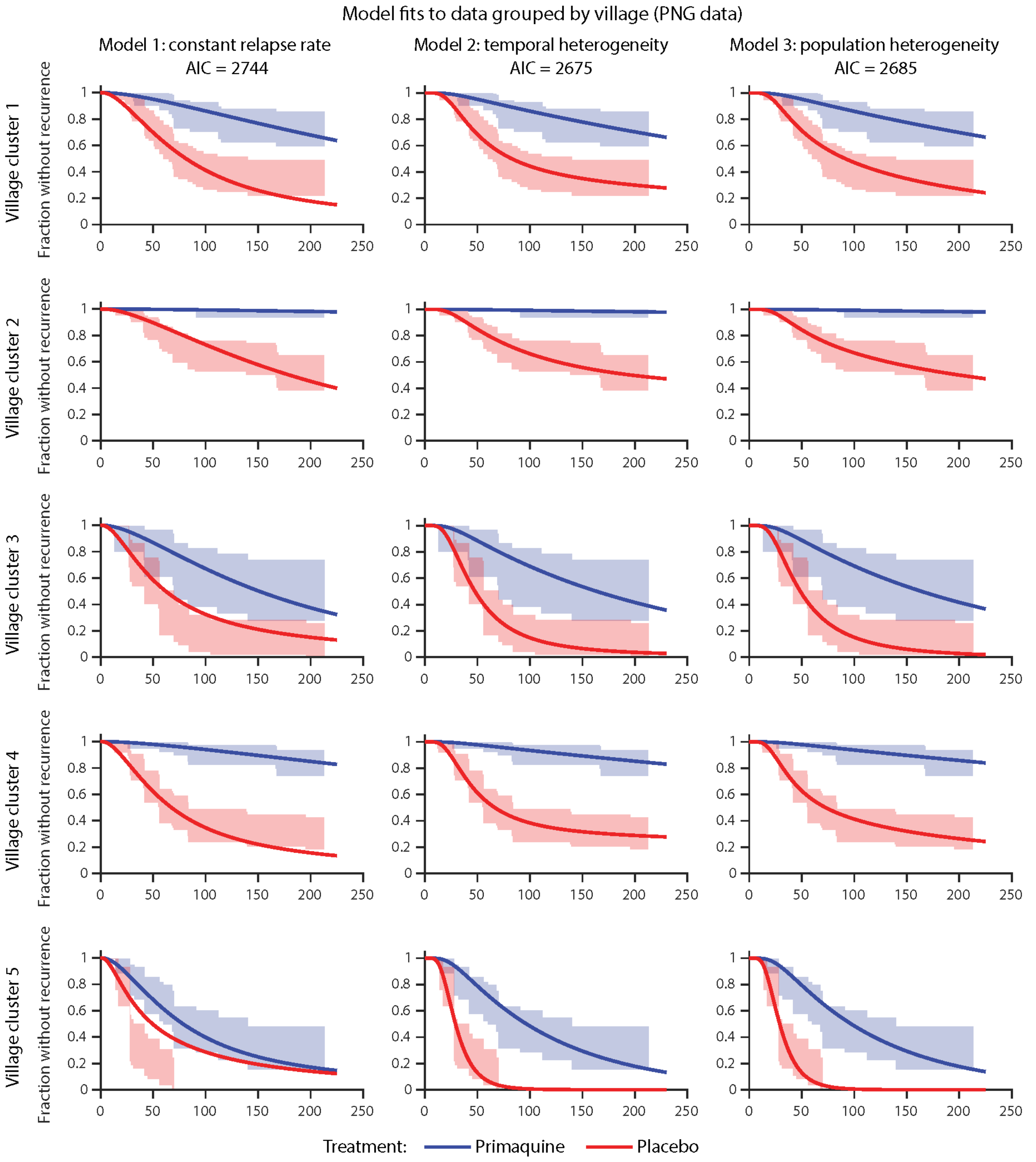

Fig C Fit of models 1 to 3 to the time to first P vivax infection by PCR in the PNG data grouped by village. All villages were fit simultaneously with the same drug washout time distribution, the rate of new infections and relapses were allowed to vary between villages. The lines indicate the model fit and the shaded area the 95% confidence region from the data. For the parameters of the model fit see **Tables E**, **F** and **G** in **S1 Tables**.

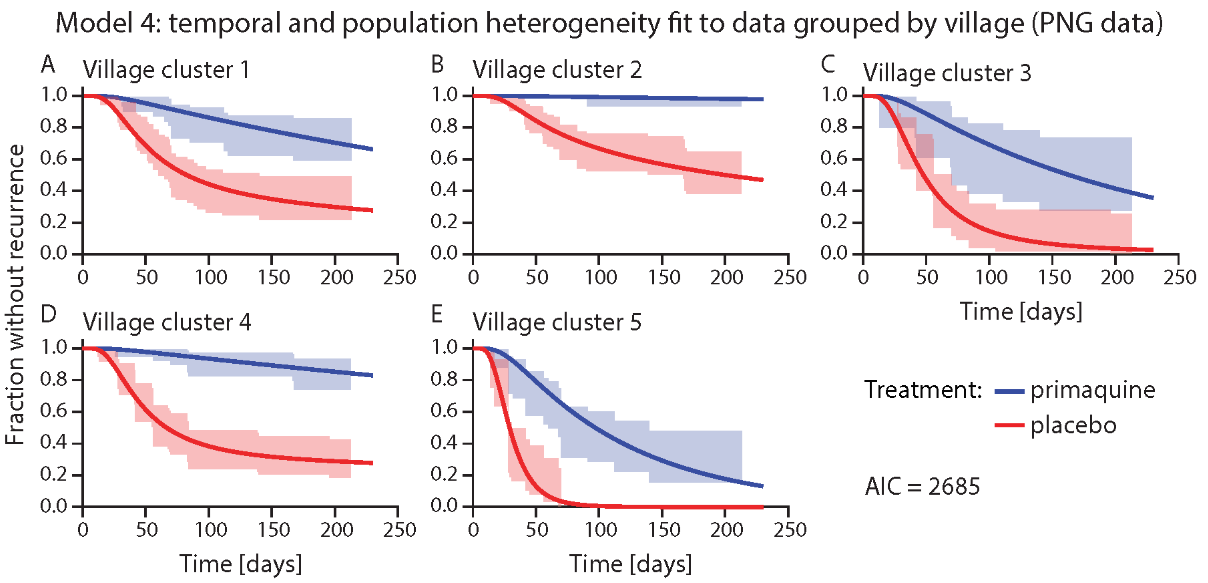

Fig D Fit of the temporal and population heterogeneity model to the first P vivax infection by PCR in the PNG data grouped by village. All villages were fit simultaneously with the same drug washout time distribution, the rate of new infections and relapses were allowed to vary between villages. The lines indicate the model fit and the shaded area the 95% confidence region from the data. For the parameters of the model fit see **Table H** in **S1 Tables**.

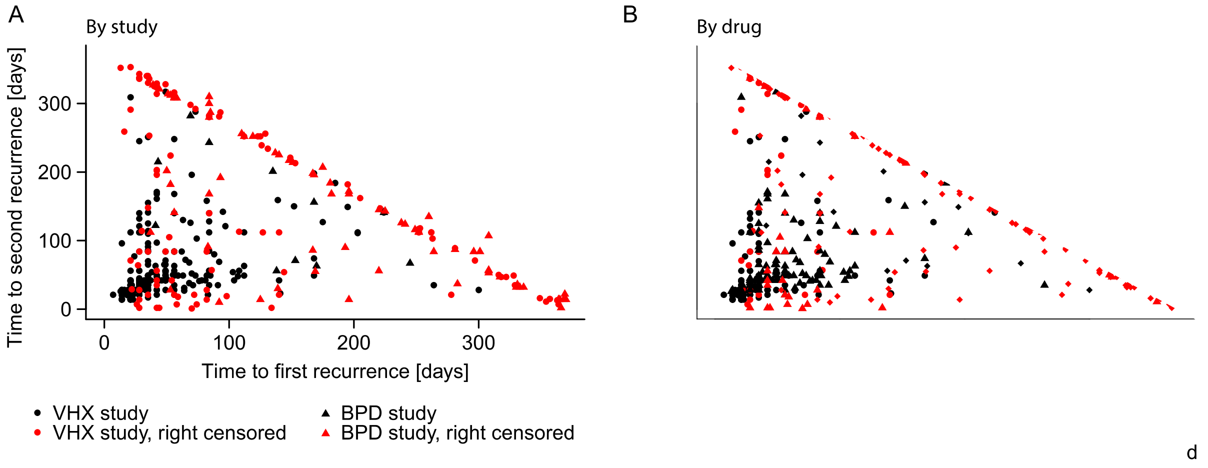

Fig E Association between time to first recurrence and time from first to second recurrence in the Thailand-Myanmar data. Different symbols represent different studies. (B) The symbols represent the different drugs. The Spearman correlation between time to first recurrence and time from first to second recurrence can be found in **Table K** in **S1 Tables**.

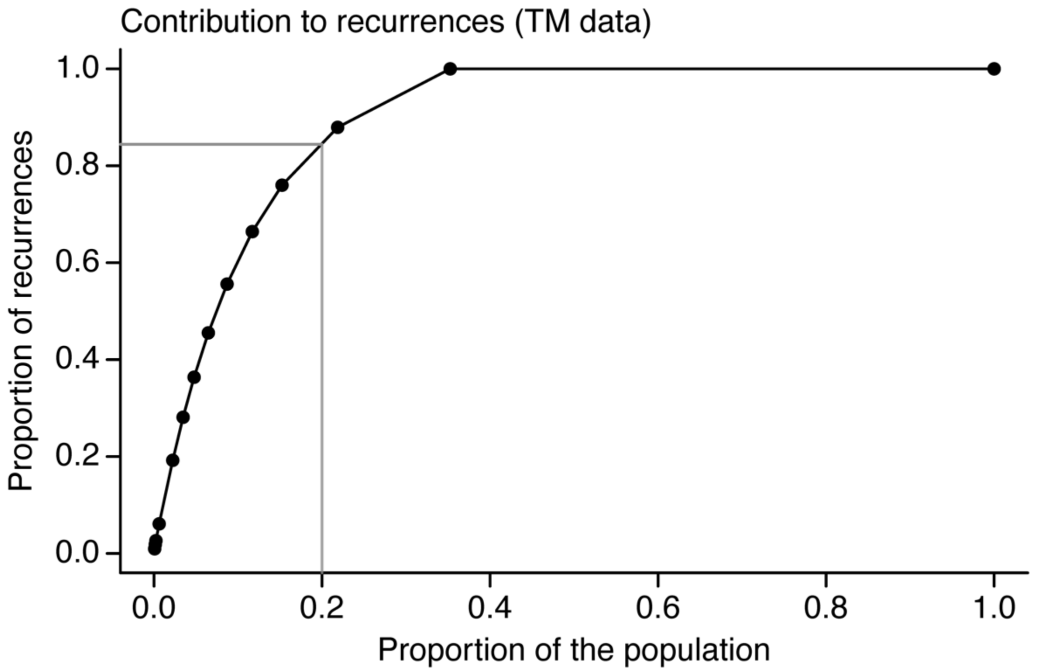

Fig F Contribution to recurrences for all patients in the Thailand-Myanmar data. This figure shows which percent of recurrences is caused by which percent of the population. Each dot represents the number of recurrences from 14 to 0 (from left to right), i.e., the first dot represents the percent of the population with at least 14 recurrences (x-axis) and the percent of recurrences caused by the patients with at least 14 recurrences (y-axis). The 20% of the population with the highest number of recurrences cause almost 85% of all recurrences (gray lines). This figure includes all patients, i.e., those who were treated for blood-stage infections only and those who were treated for both blood- and liver-stage infections. For the contribution to relapses for patients who were treated only for blood-stage infections see **Fig. 6**A.

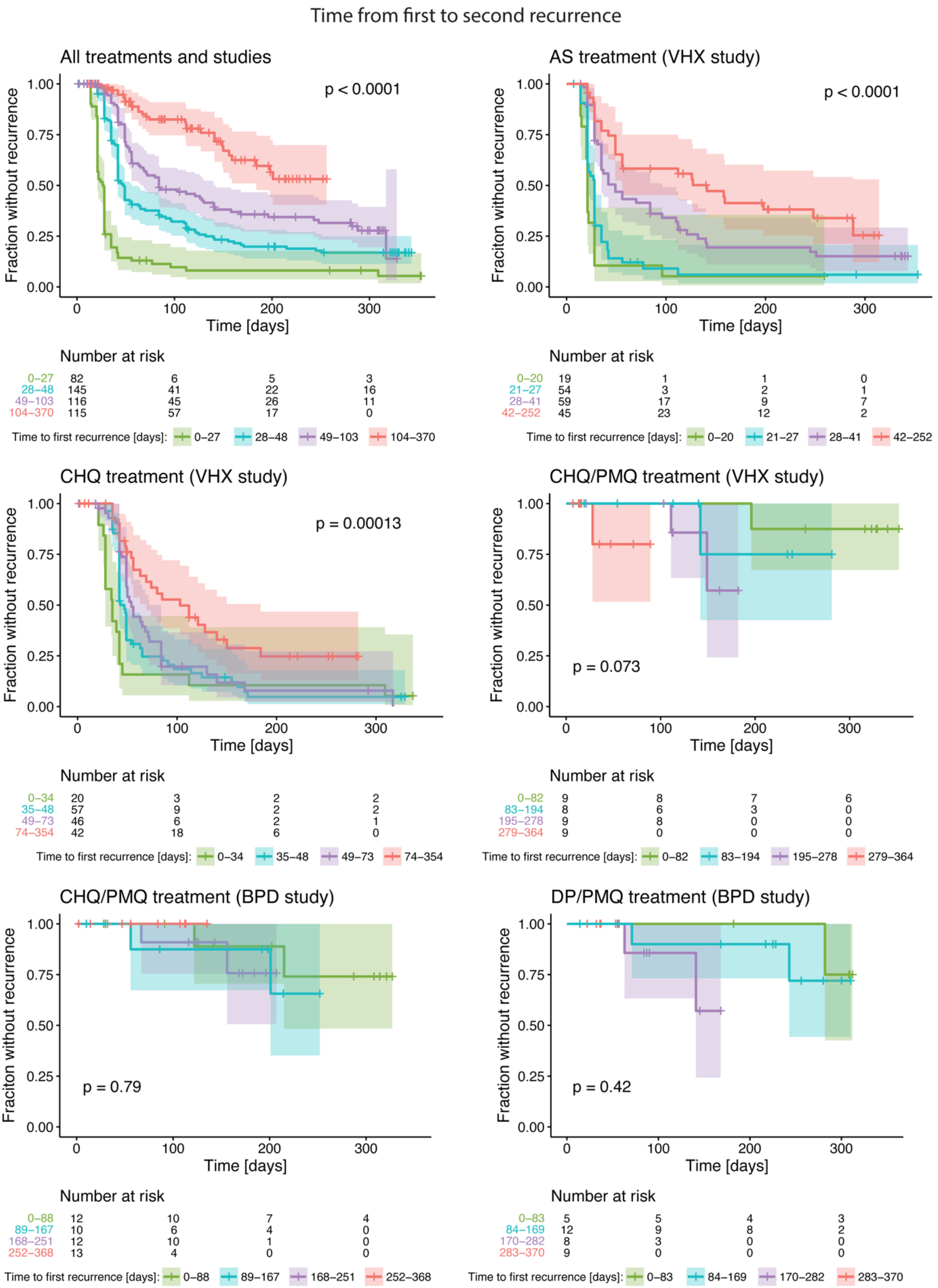

Fig G Time from first to second recurrence by the time to first recurrence quartiles for the different antimalarial treatments and studies in the Thailand-Myanmar data. The shaded areas are the 95% confidence regions for the survival curves and the p-value of the log-rank test for the comparison of the survival curves is shown in each panel.

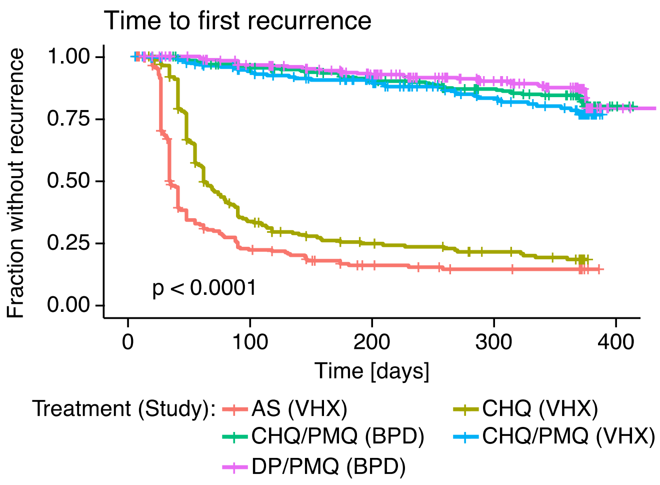

Fig H Time to first recurrence for the Thailand-Myanmar data grouped by antimalarial treatment and study. The p-value of the log-rank test for the comparison of all survival curves is shown in the lower left corner of the plot. The survival curves are significantly different, however, there is no significant difference between the survival curves of individuals treated with primaquine (CHQ/PMQ and DP/PMQ; p-value 0.074). The artesunate (AS) and chloroquine (CHQ) treatment survival curves are significantly different (p-value <0.0001). This difference is due to the different dynamics within the first 50 days as excluding recurrences and censoring within the first 50 days gives a non-significant p-value of 0.14.

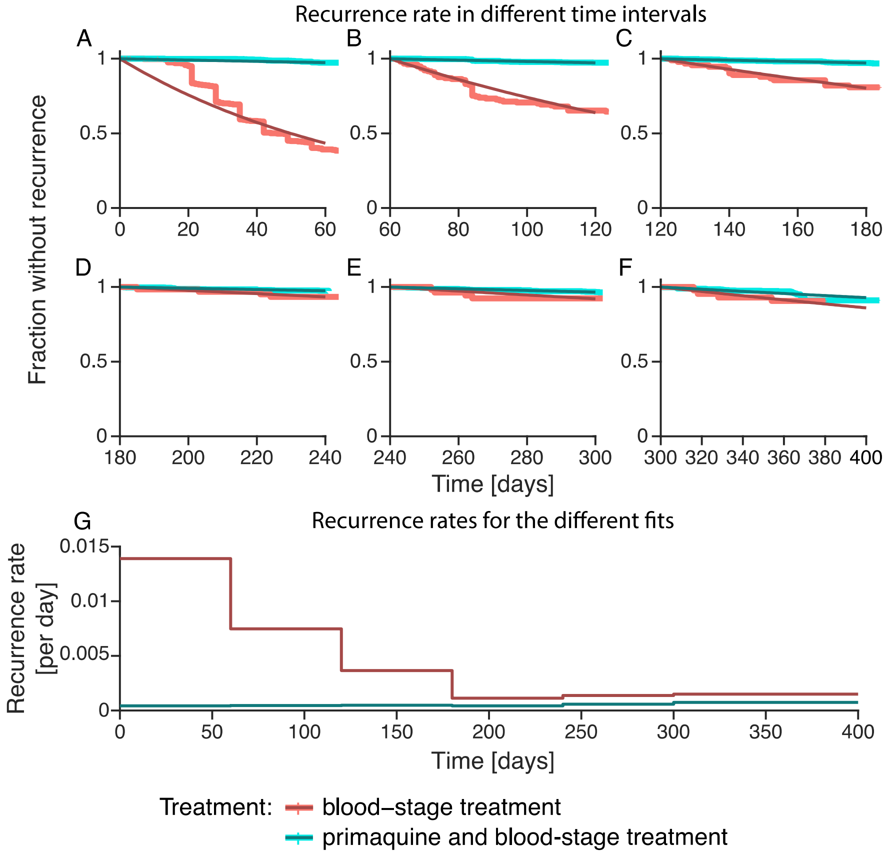

Fig I Recurrence rate on days 0-60, 60-120, 120-180. 180-240, 240-300, and 300-400 in the Thailand-Myanmar data. (A-F) Model fit to a part of the data grouped by blood-stage treatment and primaquine and blood-stage treatment. The data are shown in the lighter color, the model fits in a darker color. (G) Recurrence rates for the two different treatment groups and the fits shown in (A-F) over time. The estimated percentage of blood-stage infections that are relapses are 96.9%, 93.8%, 86.9%, 62.7%, 57.9%, and 50.8% for the fit to days 0-60, 60-120, 120-180. 180-240, 240-300, and 300-400, respectively.

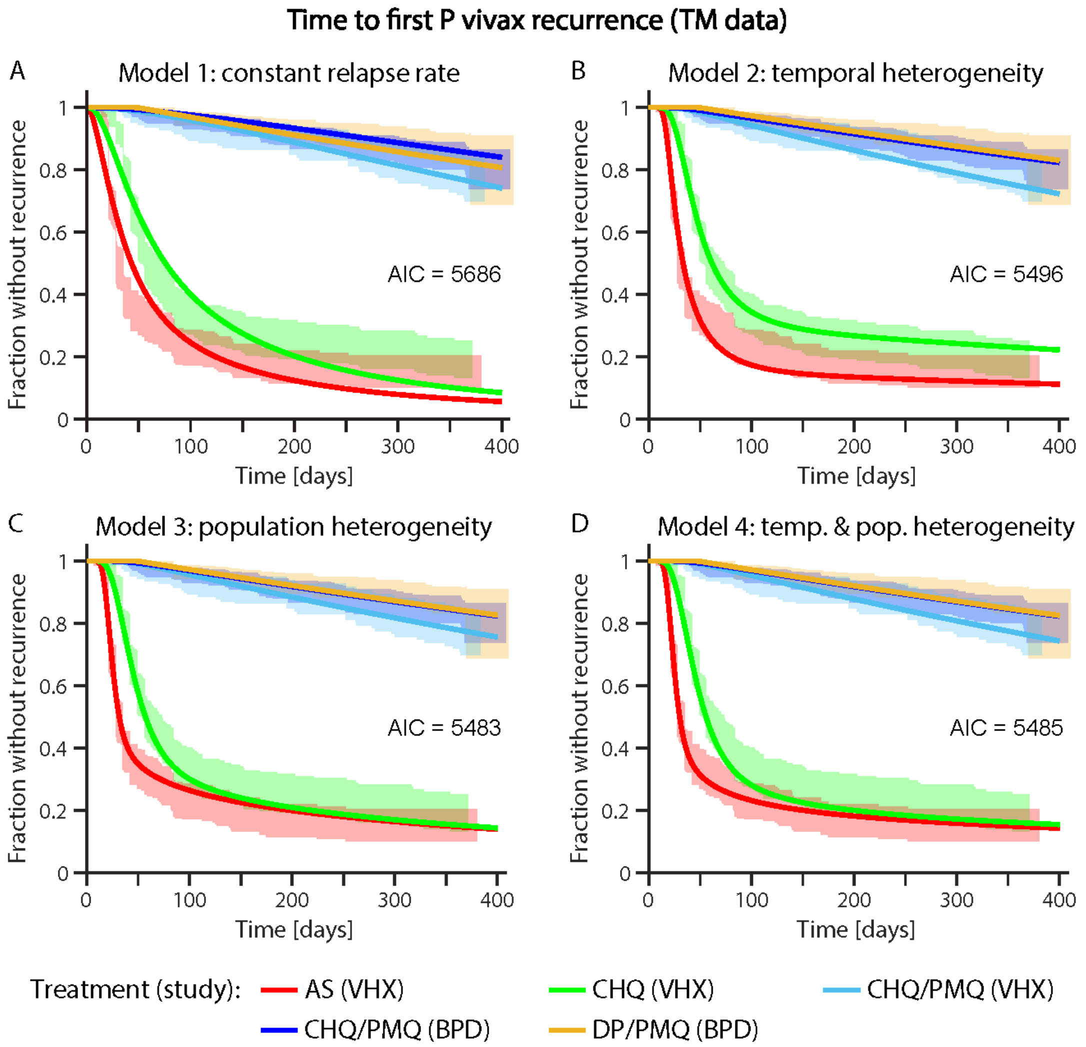

Fig J Model fit of the constant relapse rate, temporal heterogeneity, the population heterogeneity, and the temporal & population heterogeneity model to the first recurrence time in the Thailand-Myanmar data. The shaded areas are the 95% confidence regions from the data. Abbreviations: AS artesunate, CHQ chloroquine, CHQ/PMQ chloroquine and primaquine, DP/PMQ dihydroartemisinin-piperaquine and primaquine, VHX Vivax History study, BPD best Primaquine Dose study. For the parameters of the model fit see **Tables N**, **O**, **P** and **Q** in **S1 Tables**.

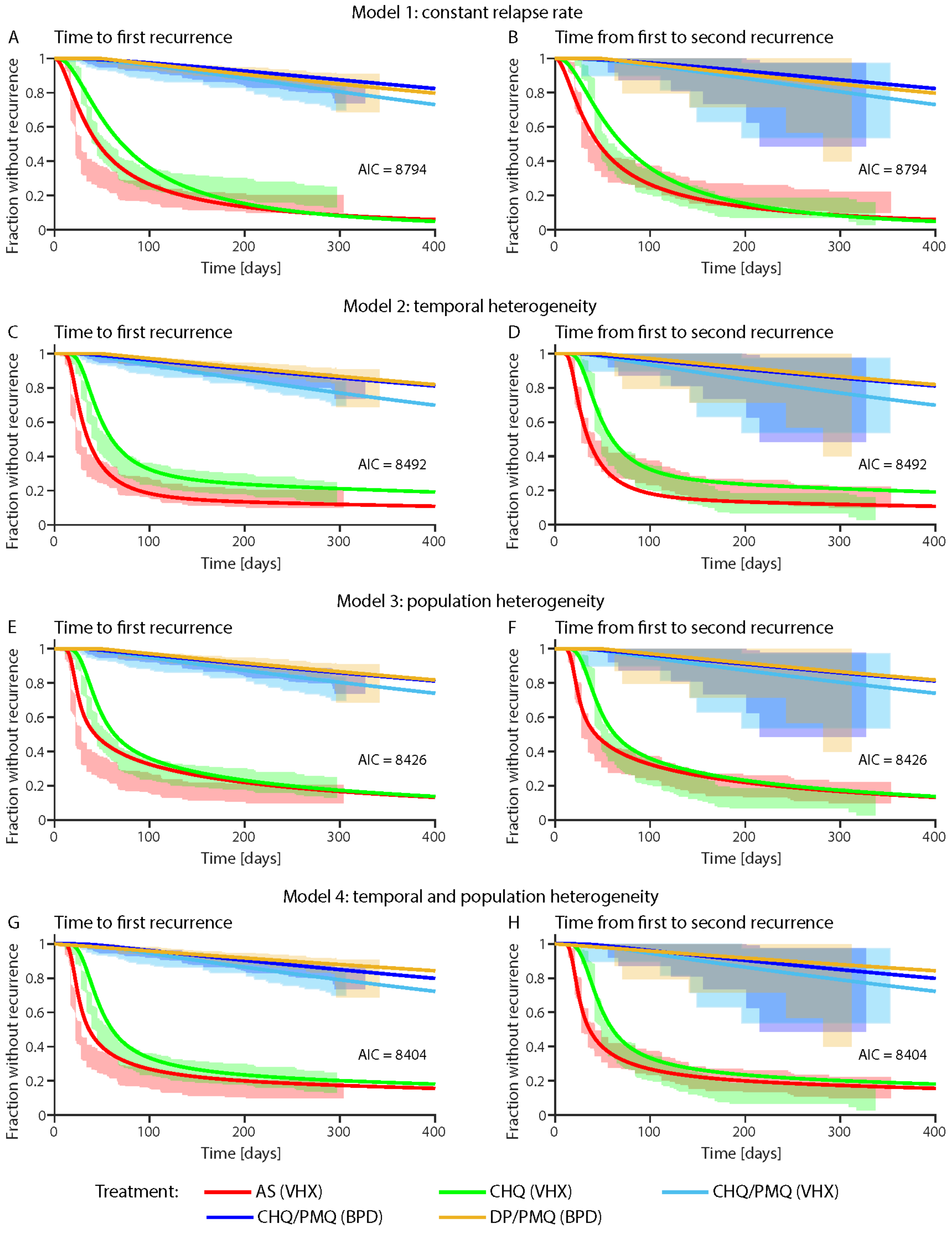

Fig K Model fit of the constant relapse rate, temporal heterogeneity, the population heterogeneity, and the temporal and population heterogeneity model to the first and second recurrence times in the Thailand-Myanmar data simultaneously. The shaded areas are the 95% confidence regions from the data. The comparison of models 3 and 4 with the likelihood-ratio test indicates that model 4 fits the data significantly better than model 3 (p-value < 0.0001). Abbreviations: AS artesunate, CHQ chloroquine, CHQ/PMQ chloroquine and primaquine, DP/PMQ dihydroartemisinin-piperaquine and primaquine, VHX Vivax History study, BPD best Primaquine Dose study. For the parameters of the model fit see **Tables R**, **S**, **T** and **U** in **S1 Tables**.

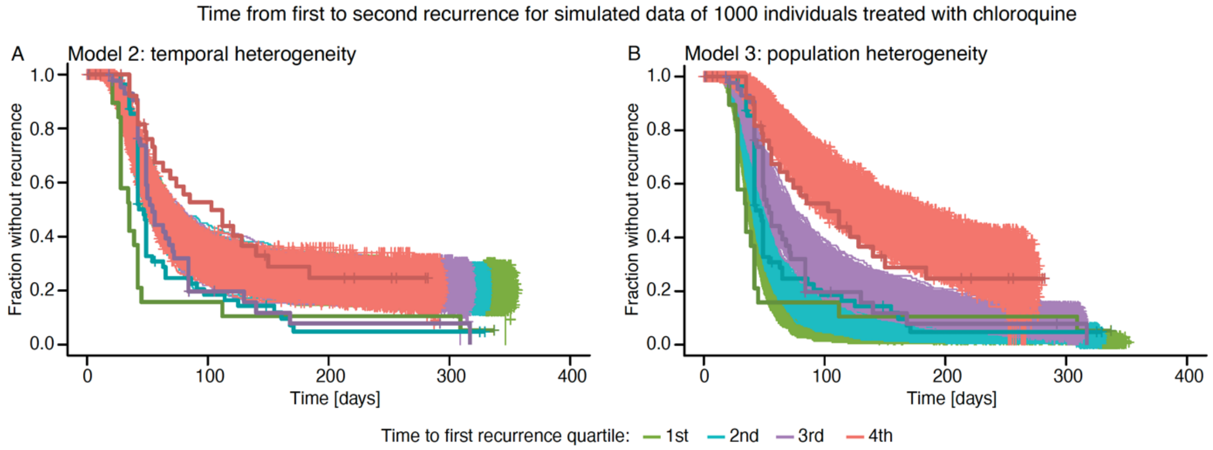

Fig L Comparison of simulated data for models 2 and 3 with the original data from the Thailand-Myanmar border region. This figure shows the survival curves of 1000 simulated populations of 1000 simulated individuals with chloroquine treatment (thin lines). The survival curves from the original data are shown for comparison as bold and darker lines. Both the simulated data and the original data are grouped by the time to first recurrence quartiles. The parameters used for this simulation are the parameter values of model fits to the TM data 1st and 2nd recurrence (see **Tables S** and **T** in **S1 Tables**). (A) Data simulated using the temporal heterogeneity model. (B) Data simulated using the population heterogeneity model.

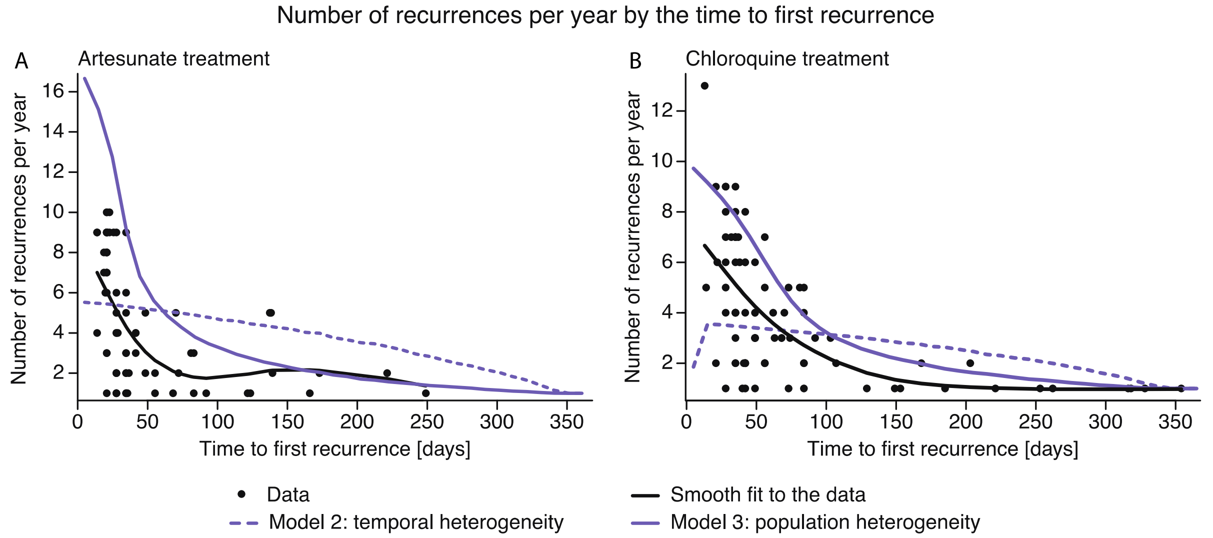

Fig M Number of recurrences per year in the Thailand-Myanmar data and simulated data for artesunate (A) and chloroquine (B) treatment. Each dot represents an individual from the data who had an overall follow-up time of at least 1 year. Patients with a follow-up less than one year were excluded. The smooth fit to the data is a smooth spline (fit using the "smooth.spline" function in R with 4 degrees of freedom). The number of recurrences per year for the models were calculated from the simulated data as the average number of recurrences over all individuals with time to first recurrence within the same 10-day time interval.
