# Supplementary material for: Population heterogeneity in Plasmodium vivax relapse risk
Source: PLoS Negl Trop Dis. 2022 Dec 19;16(12):e0010990. doi: 10.1371/journal.pntd.0010990 (PMC9810152; doi:10.1371/journal.pntd.0010990)
Supplement: S1 Tables — (PDF) [file pntd.0010990.s002.pdf]

S1 Tables

Parameters of model 1: constant relapse rate (PNG data)

Parameter	MLE	95% CI
Mean of the logarithmic values of the drug washout time distribution	3.97	[3.72,4.23]
Standard deviation of the logarithmic values of the drug washout time distribution	1.81	[1.47,2.24]
Recurrence rate for patients treated only for blood-stage infections [per day]	0.044	[0.035,0.056]
Recurrence rate for patients treated with primaquine [per day]	0.0035	[0.0025,0.0050]

Table A Maximum likelihood estimates of the parameters of the first model fit to the first recurrence time in the PNG data. Abbreviations: MLE Maximum Likelihood Estimate, CI Confidence Interval.

Parameters of model 2: temporal heterogeneity (PNG data)

Parameter	MLE	95% CI
Mean of the logarithmic values of the drug washout time distribution	3.01	[2.75, 3.24]
Standard deviation of the logarithmic values of the drug washout time distribution	0.46	[0.36,0.54]
Rate of new infections [per day]	0.0019	[0.0015,0.0024]
Initial relapse rate of patients [per day]	0.033	[0.020,0.063]
Exponential decay rate of the relapse rate [per day]	0.022	[0.015,0.030]

Table B Maximum likelihood estimates of the parameters of the second model fit to the first recurrence time in the PNG data. Abbreviations: MLE Maximum Likelihood Estimate, CI Confidence Interval.

Parameters of model 3: population heterogeneity (PNG data)

Parameter	MLE	95% CI
Mean of the logarithmic values of the drug washout time distribution	3.11	[2.83,3.65]
Standard deviation of the logarithmic values of the drug washout time distribution	0.48	[0.38,0.55]
Rate of new infections [per day]	0.0019	[0.0015,0.0025]
Mean of the logarithmic values of the relapse rate distribution	-4.73	[-5.29,45.39]
Standard deviation of the logarithmic values of the relapse rate distribution	2.26	[1.48,389.9]

Table C Maximum likelihood estimates of the parameters of the third model fit to the first recurrence time in the PNG data. Abbreviations: MLE Maximum Likelihood Estimate, CI Confidence Interval.

Parameters of model 4: temporal and population heterogeneity (PNG data)

Parameter	MLE	95% CI
Mean of the logarithmic values of the drug washout time distribution	3.01	[2.78,3.23]
Standard deviation of the logarithmic values of the drug washout time distribution	0.46	[0.36,0.54]
Rate of new infections [per day]	0.0019	[0.0015,0.0024]
Mean of the logarithmic values of the initial relapse rate distribution	-3.40	[-3.87,-2.84]
Standard deviation of the logarithmic values of the initial relapse rate distribution	0.0040	[0.0018,0.11]
Exponential decay rate of the relapse rate [per day]	0.022	[0.015,0.030]

Table D Maximum likelihood estimates of the parameters of the third model fit to the first recurrence time in the PNG data. Abbreviations: MLE Maximum Likelihood Estimate, CI Confidence Interval.

Parameters of model 1: constant relapse rate (PNG data by village)

	Parameter	MLE	95% CI
All villages	Mean of the logarithmic values of the drug washout time distribution	3.7	[2.65,4.00]
	Standard deviation of the logarithmic values of the drug washout time distribution	1.44	[0.28,1.71]
Village 1	Recurrence rate for placebo group [per day]	0.028	[0.0055,0.047]
	Recurrence rate for primaquine group [per day]	0.0031	[0.0013,0.0050]
Village 2	Recurrence rate for placebo group [per day]	0.007	[0.0031,0.014]
	Recurrence rate for primaquine group [per day]	0.00014	[0.00,0.0005]
Village 3	Recurrence rate for placebo group [per day]	0.055	[0.018,0.0078]
	Recurrence rate for primaquine group [per day]	0.0092	[0.0026,0.19]
Village 4	Recurrence rate for placebo group [per day]	0.044	[0.0068,0.066]
	Recurrence rate for primaquine group [per day]	0.0013	[0.005,0.0023]
Village 5	Recurrence rate for placebo group [per day]	0.12	[0.054,0.19]
	Recurrence rate for primaquine group [per day]	0.031	[0.0062,0.066]

Table E Maximum likelihood estimates of the parameters of the constant relapse rate model fit to the first recurrence time in the PNG data with all villages fit simultaneously with the same drug washout time distribution. Abbreviations: MLE Maximum Likelihood Estimate, CI Confidence Interval.

Parameters of model 2: temporal heterogeneity (PNG data by village)

	Parameter	MLE	95% CI
All villages	Mean of the logarithmic values of the drug washout time distribution	3.15	[2.97,3.36]
	Standard deviation of the logarithmic values of the drug washout time distribution	0.49	[0.38,0.51]
Village 1	Rate of new infections [per day]	0.0020	[0.0012,0.0031]
	Initial relapse rate of patients [per day]	0.027	[0.014,0.059]
	Exponential decay rate of the relapse rate [per day]	0.019	[0.011,0.030]
Village 2	Rate of new infections [per day]	0.00011	[1.42×10^{-10},0.00036]
	Initial relapse rate of patients [per day]	0.0090	[0.0042,0.019]
	Exponential decay rate of the relapse rate [per day]	0.0080	[0.0011,0.017]
Village 3	Rate of new infections [per day]	0.0051	[0.0024,0.0094]
	Initial relapse rate of patients [per day]	0.043	[0.020,0.11]
	Exponential decay rate of the relapse rate [per day]	0.011	[3.06×10^{-7},0.027]
Village 4	Rate of new infections [per day]	0.00091	[0.00041,0.0015]
	Initial relapse rate of patients [per day]	0.044	[0.022,0.12]
	Exponential decay rate of the relapse rate [per day]	0.022	[0.013,0.037]
Village 5	Rate of new infections [per day]	0.010	[0.0064,0.016]
	Initial relapse rate of patients [per day]	0.24	[0.068,12.59]
	Exponential decay rate of the relapse rate [per day]	0.015	[4.20×10^{-7},0.045]

Table F Maximum likelihood estimates of the parameters of the temporal heterogeneity model fit to the first recurrence time in the PNG data with all villages fit simultaneously with the same drug washout time distribution. Abbreviations: MLE Maximum Likelihood Estimate, CI Confidence Interval.

Parameters of model 3: population heterogeneity (PNG data by village)

	Parameter	MLE	95% CI
All villages	Mean of the logarithmic values of the drug washout time distribution	3.2	[2.91,3.45]
	Standard deviation of the logarithmic values of the drug washout time distribution	0.49	[0.38,0.54]
Village 1	Rate of new infections [per day]	0.0021	[0.0012,0.0034]
	Mean of the logarithmic values of the relapse rate distribution	-5.03	[-6.45,-4.18]
	Standard deviation of the logarithmic values of the relapse rate distribution	1.85	[1.04,4.34]
Village 2	Rate of new infections [per day]	0.00011	[3.37×10^{-11},0.00036]
	Mean of the logarithmic values of the relapse rate distribution	-5.69	[-6.68,-5.04]
	Standard deviation of the logarithmic values of the relapse rate distribution	1.63	[0.51,3.12]
Village 3	Rate of new infections [per day]	0.0051	[0.0024,0.010]
	Mean of the logarithmic values of the relapse rate distribution	-3.57	[-4.56,-2.53]
	Standard deviation of the logarithmic values of the relapse rate distribution	0.94	[0.0015,2.31]
Village 4	Rate of new infections [per day]	0.00088	[0.00035,0.0015]
	Mean of the logarithmic values of the relapse rate distribution	-4.39	[-5.41,-3.24]
	Standard deviation of the logarithmic values of the relapse rate distribution	2.26	[1.19,5.21]
Village 5	Rate of new infections [per day]	0.01	[0.0060,0.016]
	Mean of the logarithmic values of the relapse rate distribution	-1.24	[-2.83,3.52×10^{6}]
	Standard deviation of the logarithmic values of the relapse rate distribution	1.13	[0.0011,9.66×10^{5}]

Table G Maximum likelihood estimates of the parameters of the population heterogeneity model fit to the first recurrence time in the PNG data with all villages fit simultaneously with the same drug washout time distribution. Abbreviations: MLE Maximum Likelihood Estimate, CI Confidence Interval.

Parameters of model 4: temporal and population heterogeneity (PNG data by village)

	Parameter	MLE	95% CI
All villages	Mean of the logarithmic values of the drug washout time distribution	3.18	[2.94,3.40]
	Standard deviation of the logarithmic values of the drug washout time distribution	0.51	[0.39,0.56]
Village 1	Rate of new infections [per day]	0.0020	[0.0011,0.0032]
	Mean of the logarithmic values of the initial relapse rate distribution	-3.56	[-4.30,-2.82]
	Standard deviation of the logarithmic values of the initial relapse rate distribution	0.015	[0.0010,0.33]
	Exponential decay rate of the relapse rate [per day]	0.019	[0.010,0.030]
Village 2	Rate of new infections [per day]	0.00011	[1.53×10^{-10},0.0004]
	Mean of the logarithmic values of the initial relapse rate distribution	-5.61	[-6.30,-3.97]
	Standard deviation of the logarithmic values of the initial relapse rate distribution	1.56	[0.0073,2.26]
	Exponential decay rate of the relapse rate [per day]	0.00074	[8.74×10^{-8},0.017]
Village 3	Rate of new infections [per day]	0.0051	[0.0022,0.010]
	Mean of the logarithmic values of the initial relapse rate distribution	-3.08	[-4.05,-2.06]
	Standard deviation of the logarithmic values of the initial relapse rate distribution	0.038	[0.0009,1.11]
	Exponential decay rate of the relapse rate [per day]	0.011	[1.49×10^{-7},0.028]
Village 4	Rate of new infections [per day]	0.00092	[0.0004,0.0016]
	Mean of the logarithmic values of the initial relapse rate distribution	-3.07	[-3.97,-2.24]
	Standard deviation of the logarithmic values of the initial relapse rate distribution	0.041	[0.0010,1.59]
	Exponential decay rate of the relapse rate [per day]	0.023	[0.0080,0.037]
Village 5	Rate of new infections [per day]	0.010	[0.0063,0.017]
	Mean of the logarithmic values of the initial relapse rate distribution	-1.36	[-2.78,2.57]
	Standard deviation of the logarithmic values of the initial relapse rate distribution	0.085	[0.0024,2.02]
	Exponential decay rate of the relapse rate [per day]	0.011	[1.68×10^{-7},0.037]

Table H Maximum likelihood estimates of the parameters of the temporal and population heterogeneity model fit to the first recurrence time in the PNG data with all villages fit simultaneously with the same drug washout time distribution. Abbreviations: MLE Maximum Likelihood Estimate, CI Confidence Interval.

Antimalarial treatments by study in the Thailand-Myanmar data

Study	**VivaX History study (VHX)**	**Best Primaquine Dose study (BPD)**
Treatment	Artesunate (AS)<br>Chloroquine (CHQ)<br>Chloroquine and primaquine (CHQ/PMQ)	Chloroquine and primaquine (CHQ/PMQ)<br>Dihydroartemisinin-piperaquine and primaquine (DP/PMQ)

Table I Antimalarial treatments by study in the Thailand-Myanmar data. In the VHX study, patients were treated with either artesunate (AS), chloroquine (CHQ), or chloroquine and primaquine (CHQ/PMQ). In the BPD study, patients were treated with chloroquine and primaquine (CHQ/PMQ) or with dihydroartemisinin-piperaquine and primaquine (DP/PMQ). These abbreviations are used through the supplement.

Contribution to recurrences by number of recurrences in the Thailand-Myanmar data

**Number of recurrences**	**Number of individuals (%)**		**Number of recurrences caused by individuals with _ recurrences (%)**	
0	841	(64.74%)	0	(0%)
1	174	(13.39%)	174	(12.07%)
2	86	(6.62%)	172	(11.94%)
3	46	(3.54%)	138	(9.58%)
4	39	(3.00%)	156	(10.83%)
5	29	(2.23%)	145	(10.06%)
6	22	(1.69%)	132	(9.16%)
7	17	(1.31%)	119	(8.26%)
8	16	(1.23%)	128	(8.88%)
9	21	(1.62%)	189	(13.12%)
10	5	(0.38%)	50	(3.47%)
11	1	(0.08%)	11	(0.76%)
12	0	(0%)	0	(0%)
13	1	(0.08%)	13	(0.90%)
14	1	(0.08%)	14	(0.97%)

Table J Contribution of individuals with different number of recurrences to the overall number of recurrences in the Thailand-Myanmar data. Data for both studies and all antimalarial treatments. This table shows, e.g., that the individuals with 3 or more recurrences are 15.2% of the population but they cause 75.99% of all recurrences. For a visualization of the contribution to recurrences see **Fig F** in **S2 Figures**.

Spearman correlation between time to first recurrence and time from first to second recurrence in the Thailand-Myanmar data

Data	All data (p-value)		Excluding censored data (p-value)	
All data	0.33	(<0.0001*)	0.63	(<0.0001*)
VHX study	0.39	(<0.0001*)	0.62	(<0.0001*)
BPD study	-0.69	(<0.0001*)	-0.54	(0.09)
AS treated	0.53	(<0.0001*)	0.59	(<0.0001*)
CHQ treated	0.31	(<0.0001*)	0.42	(<0.0001*)
PMQ+ treated	-0.73	(<0.0001*)	-0.63	(0.01)

Table K Spearman correlation between time to first recurrence and time from first to second recurrence in the Thailand-Myanmar data. In the VHX study individuals were treated either with artesunate (AS), chloroquine (CHQ), or chloroquine and primaquine (PMQ+) and in the BPD study individuals were treated with chloroquine and primaquine or with dihydroartemisinin-piperaquine and primaquine (PMQ+). The asterisk (*) indicates that the exact p-value could not be computed in R due to ties.

Spearman correlation between time to first recurrence and time from first to second recurrence with recurrence times restricted to 182 days

	Artesunate (p-value)		Chloroquine (p-value)	
Model 1: constant relapse rate	0.0008	(0.52)	0.0009	(0.48)
Model 2: temporal heterogeneity	-0.001	(0.39)	0.002	(0.079)
Model 3: population heterogeneity	0.52	(<0.0001)	0.38	(<0.0001)
Thailand-Myanmar data	0.55	(<0.0001)	0.46	(<0.0001)

Table L Spearman correlation between time to first recurrence and time from first to second recurrence in the simulated data and the TM data excluding censored data with recurrence times restricted to 182 days. All of the 1,000,000 simulated individuals who had at least two recurrences during the 1-year-simulation with both recurrences within the 182 days of the previous recurrence were used to compute the Spearman correlation. For the TM data, we show here the Spearman correlation for all individuals who had at least two known recurrences and each recurrence was within 182 days of the last known recurrence. The recurrence times were restricted to 182 days to avoid a bias in the second recurrence time due to the first recurrence time that is present if the sum of both recurrence times is restricted to be at most 365 days (e.g., if an individual has a long time to the first recurrence, then the time to the second recurrence is necessarily short, however if we restrict both recurrence times to 182 days, then the first recurrence time does not give any information about the second recurrence which may be at any time between 1 and 182 days after the first recurrence).

Cox regression on time from 1st to 2nd recurrence in the Thailand-Myanmar data

	**AS treatment**	**CHQ treatment**
Hazard ratio	0.987	0.989
p-value	0.0001	0.00004
95% CI	0.9805-0.9937	0.9833-0.994

Table M Cox regression of the time from first to second recurrence with time to first recurrence as a continuous variable for individuals treated with artesunate (AS) or chloroquine (CHQ) in the Thailand-Myanmar data. The hazard ratio shows that individuals with a longer time to the first recurrence have a lower risk of recurrence, i.e., they also have a longer time to their second recurrence.

Parameters of model 1: constant relapse rate (for fitting to the first recurrence time in the Thailand-Myanmar data)

Parameter	MLE	95% CI
Mean of the logarithmic values of the drug washout time distribution for AS	3.26	[3.08,3.46]
Standard deviation of the logarithmic values of the drug washout time distribution for AS	1.69	[1.33,2.15]
Mean of the logarithmic values of the drug washout time distribution for CHQ	4.10	[3.95,4.32]
Standard deviation of the logarithmic values of the drug washout time distribution for CHQ	1.35	[1.15,1.61]
Mean of the logarithmic values of the drug washout time distribution for DP	3.88	[3.86,6.27]
Standard deviation of the logarithmic values of the drug washout time distribution for DP	0.057	[0.052,4.34]
Recurrence rate for patients treated only for blood-stage infections (VHX study) [per day]	0.0721	[0.0606,0.0893]
Recurrence rate for patients treated with primaquine in the VHX study [per day]	0.00106	[0.0007,0.0015]
Recurrence rate for patients treated with primaquine in the BPD study [per day]	0.00061	[0.0005,0.001]

Table N Maximum likelihood estimates of the parameters of the first model fit to the first recurrence time in the Thailand-Myanmar data. Abbreviations: MLE Maximum Likelihood Estimate, CI Confidence Interval, AS artesunate treatment, CHQ chloroquine treatment, DP dihydroartemisinin-piperaquine treatment, VHX Vivax History study, BPD Best Primaquine Dose study.

Parameters of model 2: temporal heterogeneity (for fitting to the first recurrence time in the Thailand-Myanmar data)

Parameter	MLE	95% CI
Mean of the logarithmic values of the drug washout time distribution for AS	2.81	[2.74,2.88]
Standard deviation of the logarithmic values of the drug washout time distribution for AS	0.26	[0.15,0.34]
Mean of the logarithmic values of the drug washout time distribution for CHQ	3.42	[3.34,3.51]
Standard deviation of the logarithmic values of the drug washout time distribution for CHQ	0.38	[0.28,0.46]
Mean of the logarithmic values of the drug washout time distribution for DP	3.89	[3.86,3.94]
Standard deviation of the logarithmic values of the drug washout time distribution for DP	0.057	[0.053,0.063]
Rate of new infections in the VHX study [per day]	0.00089	[0.0007,0.0011]
Rate of new infections in the BPD study [per day]	0.00053	[0.0004,0.0006]
Initial relapse rate for patients treated with AS or CHQ only (VHX study) [per day]	0.088	[0.0682,0.12]
Exponential decay rate of the relapse rate [per day]	0.029	[0.025,0.035]

Table O Maximum likelihood estimates of the parameters of the second model fit to the first recurrence time in the Thailand-Myanmar data. Abbreviations: MLE Maximum Likelihood Estimate, CI Confidence Interval, AS artesunate treatment, CHQ chloroquine treatment, DP dihydroartemisinin-piperaquine treatment, VHX Vivax History study, BPD Best Primaquine Dose study.

Parameters of model 3: population heterogeneity (for fitting to the first recurrence time in the Thailand-Myanmar data)

Parameter	MLE	95% CI
Mean of the logarithmic values of the drug washout time distribution for AS	3.11	[3.05,3.18]
Standard deviation of the logarithmic values of the drug washout time distribution for AS	0.28	[0.21,0.34]
Mean of the logarithmic values of the drug washout time distribution for CHQ	3.68	[3.61,3.77]
Standard deviation of the logarithmic values of the drug washout time distribution for CHQ	0.42	[0.35,0.49]
Mean of the logarithmic values of the drug washout time distribution for DP	3.90	[1.88,4.76]
Standard deviation of the logarithmic values of the drug washout time distribution for DP	0.063	[0.057,20.42]
Rate of new infections in the VHX study [per day]	0.00078	[0.0006,0.001]
Rate of new infections in the BPD study [per day]	0.00054	[0.0005,0.0007]
Mean of the logarithmic values of the relapse rate distribution for AS and CHQ (VHX study)	-1.88	[-2.66,-0.42]
Standard deviation of the logarithmic values of the relapse rate distribution for AS and CHQ (VHX study)	5.05	[3.79,7.50]

Table P Maximum likelihood estimates of the parameters of the third model fit to the first recurrence time in the Thailand-Myanmar data. Abbreviations: MLE Maximum Likelihood Estimate, CI Confidence Interval, AS artesunate treatment, CHQ chloroquine treatment, DP dihydroartemisinin-piperaquine treatment, VHX Vivax History study, BPD Best Primaquine Dose study.

Parameters of model 4: temporal and population heterogeneity (for fitting to the first recurrence time in the Thailand-Myanmar data)

Parameter	MLE	95% CI
Mean of the logarithmic values of the drug washout time distribution for AS	3.09	[3.03,3.14]
Standard deviation of the logarithmic values of the drug washout time distribution for AS	0.27	[0.21,0.36]
Mean of the logarithmic values of the drug washout time distribution for CHQ	3.68	[3.60,3.78]
Standard deviation of the logarithmic values of the drug washout time distribution for CHQ	0.42	[0.33,0.51]
Mean of the logarithmic values of the drug washout time distribution for DP	3.87	[3.84,3.95]
Standard deviation of the logarithmic values of the drug washout time distribution for DP	0.062	[0.056,0.068]
Rate of new infections in the VHX study [per day]	0.00083	[0.0006,0.0011]
Rate of new infections in the BPD study [per day]	0.00055	[0.0004,0.0007]
Mean of the logarithmic values of the initial relapse rate distribution for AS and CHQ (VHX study)	-1.60	[-1.92,-1.41]
Standard deviation of the logarithmic values of the initial relapse rate distribution for AS and CHQ (VHX)	3.93	[3.67,4.10]
Exponential decay rate of the relapse rate [per day]	0.0081	[0.0051,0.013]

Table Q Maximum likelihood estimates of the parameters of the fourth model fit to the first recurrence time in the Thailand-Myanmar data. Abbreviations: MLE Maximum Likelihood Estimate, CI Confidence Interval, AS artesunate treatment, CHQ chloroquine treatment, DP dihydroartemisinin-piperaquine treatment, VHX Vivax History study, BPD Best Primaquine Dose study.

Parameters of model 1: constant relapse rate (Thailand-Myanmar data)

Parameter	MLE	95% CI
Mean of the logarithmic values of the drug washout time distribution for AS	3.44	[3.29,3.62]
Standard deviation of the logarithmic values of the drug washout time distribution for AS	1.61	[1.40,1.87]
Mean of the logarithmic values of the drug washout time distribution for CHQ	4.05	[3.92,4.20]
Standard deviation of the logarithmic values of the drug washout time distribution for CHQ	1.15	[1.02,1.29]
Mean of the logarithmic values of the drug washout time distribution for DP	3.89	[2.07,5.81]
Standard deviation of the logarithmic values of the drug washout time distribution for DP	0.057	[0.052,16.64]
Recurrence rate for patients treated only for blood-stage infections (VHX study) [per day]	0.081	[0.072,0.095]
Recurrence rate for patients treated with primaquine in the VHX study [per day]	0.0011	[0.0007,0.0014]
Recurrence rate for patients treated with primaquine in the BPD study [per day]	0.00065	[0.0005,0.001]

Table R Estimates and 95% confidence intervals for the parameters of the first model fit simultaneously to the first and second recurrence time in the Thailand-Myanmar data. Abbreviations: MLE Maximum Likelihood Estimate, CI Confidence Interval, AS artesunate treatment, CHQ chloroquine treatment, DP dihydroartemisinin-piperaquine treatment, VHX Vivax History study, BPD Best Primaquine Dose study.

Parameters of model 2: temporal heterogeneity (Thailand-Myanmar data)

Parameter	MLE	95% CI
Mean of the logarithmic values of the drug washout time distribution for AS	2.79	[2.75,2.86]
Standard deviation of the logarithmic values of the drug washout time distribution for AS	0.24	[0.18,0.29]
Mean of the logarithmic values of the drug washout time distribution for CHQ	3.38	[3.33,3.45]
Standard deviation of the logarithmic values of the drug washout time distribution for CHQ	0.31	[0.26,0.36]
Mean of the logarithmic values of the drug washout time distribution for DP	3.88	[3.86,3.88]
Standard deviation of the logarithmic values of the drug washout time distribution for DP	0.057	[0.054,0.058]
Rate of new infections in the VHX study [per day]	0.00097	[0.0008,0.0012]
Rate of new infections in the BPD study [per day]	0.00057	[0.0005,0.0007]
Initial relapse rate for patients treated with AS or CHQ only (VHX study) [per day]	0.070	[0.061,0.089]
Exponential decay rate of the relapse rate [per day]	0.025	[0.022,0.029]

Table S Estimates and 95% confidence intervals for the parameters of the second model fit simultaneously to the first and second recurrence time in the Thailand-Myanmar data. Abbreviations: MLE Maximum Likelihood Estimate, CI Confidence Interval, AS artesunate treatment, CHQ chloroquine treatment, DP dihydroartemisinin-piperaquine treatment, PMQ primaquine treatment, VHX Vivax History study, BPD Best Primaquine Dose study.

Parameters of model 3: population heterogeneity (Thailand-Myanmar data)

Parameter	MLE	95% CI
Mean of the logarithmic values of the drug washout time distribution for AS	2.95	[2.90,3.00]
Standard deviation of the logarithmic values of the drug washout time distribution for AS	0.24	[0.18,0.29]
Mean of the logarithmic values of the drug washout time distribution for CHQ	3.53	[3.46,3.60]
Standard deviation of the logarithmic values of the drug washout time distribution for CHQ	0.33	[0.25,0.39]
Mean of the logarithmic values of the drug washout time distribution for DP	3.88	[-4.86,4.74]
Standard deviation of the logarithmic values of the drug washout time distribution for DP	0.060	[0.056,27.46]
Rate of new infections in the VHX study [per day]	0.0008	[0.0006,0.0011]
Rate of new infections in the BPD study [per day]	0.0006	[0.0005,0.0008]
Mean of the logarithmic values of the relapse rate distribution for AS and CHQ (VHX study)	-3.73	[-4.03,-3.39]
Standard deviation of the logarithmic values of the relapse rate distribution for AS and CHQ (VHX study)	2.80	[2.39,3.26]

Table T Estimates and 95% confidence intervals for the parameters of the third model fit simultaneously to the first and second recurrence time in the Thailand-Myanmar data. Abbreviations: MLE Maximum Likelihood Estimate, CI Confidence Interval, AS artesunate treatment, CHQ chloroquine treatment, DP dihydroartemisinin-piperaquine treatment, PMQ primaquine treatment, VHX Vivax History study, BPD Best Primaquine Dose study.

Parameters of model 4: temporal and population heterogeneity (Thailand-Myanmar data)

Parameter	MLE	95% CI
Mean of the logarithmic values of the drug washout time distribution for AS	2.93	[2.88,2.98]
Standard deviation of the logarithmic values of the drug washout time distribution for AS	0.24	[0.17,0.24]
Mean of the logarithmic values of the drug washout time distribution for CHQ	3.54	[3.47,3.60]
Standard deviation of the logarithmic values of the drug washout time distribution for CHQ	0.33	[0.26,0.40]
Mean of the logarithmic values of the drug washout time distribution for DP	-10.23	[-26.66,4.56]
Standard deviation of the logarithmic values of the drug washout time distribution for DP	26.62	[21.01,31.89]
Rate of new infections in the VHX study [per day]	0.00089	[0.0007,0.0011]
Rate of new infections in the BPD study [per day]	0.00062	[0.0005,0.0008]
Mean of the logarithmic values of the relapse rate distribution for AS and CHQ (VHX study)	-3.01	[-3.33,-2.68]
Standard deviation of the logarithmic values of the relapse rate distribution for AS and CHQ (VHX study)	1.86	[1.53,2.24]
Exponential decay rate of the relapse rate [per day]	0.012	[0.0083,0.017]

Table U Estimates and 95% confidence intervals for the parameters of the fourth model fit simultaneously to the first and second recurrence time in the Thailand-Myanmar data. Abbreviations: MLE Maximum Likelihood Estimate, CI Confidence Interval, AS artesunate treatment, CHQ chloroquine treatment, DP dihydroartemisinin-piperaquine treatment, PMQ primaquine treatment, VHX Vivax History study, BPD Best Primaquine Dose study.
