# Supplementary material for: Population heterogeneity in Plasmodium vivax relapse risk
Source: PLoS Negl Trop Dis. 2022 Dec 19;16(12):e0010990. doi: 10.1371/journal.pntd.0010990 (PMC9810152; doi:10.1371/journal.pntd.0010990)
Supplement: S1 Methods — (PDF) [file pntd.0010990.s003.pdf]

S3 Methods

Models

We constructed mathematical models for *P vivax* recurrences. For the scheme of the models see **Fig A**. Individuals are protected due to the prophylactic effect of antimalarial treatment at enrolment. The drug washout time is lognormally distributed and after drug washout individuals are susceptible to both new, mosquito-borne infections and relapses. All models include a constant infection rate but differ in how relapses are modelled (see also the Methods section in the main text).

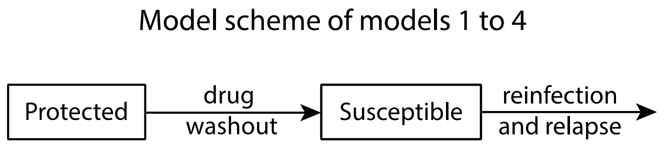

Fig A Model scheme of models 1 to 4. At enrolment patients are treated and thus protected due to the prophylactic effect of the antimalarials. After drug washout, patients are susceptible to both new infections and relapses. Models 1 to 4 all include a lognormal distributed drug washout time and a constant infection rate. The models differ in their relapse rate.

Model 1: constant relapse rate

The relapse rate is constant and the same for all individuals. Thus, the time to the next relapse is exponentially distributed and the fraction of susceptible individuals $S(t)$ at time t is given by the following ODE:

$$\frac{d}{dt} S(t) = w(t;\mu,\sigma) - (r + n)\, S(t), \qquad S(0) = 0,$$

where $w(t;\mu,\sigma)$ is the probability density function of the lognormal distribution with parameters μ and σ, r is the constant relapse rate, and n is the constant infection rate. This model equation describes that individuals become susceptible after the lognormally distributed drug washout time ($w(t;\mu,\sigma)$) and leave the compartment of susceptible individuals after a relapse occurring at rate r or a new infection occurring at rate n. Initially, all individuals are protected, thus $S(0) = 0$.

Model 2: temporal heterogeneity

The relapse rate in model 2 is a time-dependent relapse rate given by $r(t) = Ie^{-dt}$ (see main text for more details). The model equation is similar to the model equation for model 1:

$$\frac{d}{dt} S(t) = w(t;\mu,\sigma) - (r(t) + n)\, S(t), \qquad S(0) = 0,$$

where $w(t;\mu,\sigma)$ is the probability density function of the lognormal distribution with parameters μ and σ, $r(t) = Ie^{-dt}$ is the time-dependent relapse rate, and n is the constant infection rate.

Model 3: population heterogeneity

Model 3 takes population heterogeneity in relapses into account as a distribution in relapse rates. Each individual has a random relapse rate drawn from a lognormal distribution. In order

to simplify the numerical solution of model 3, we group the population into 'relapse risk groups' of equal size (see **Fig B**). As we use percentiles of the relapse risk distribution to define the relapse risk groups, all relapse risk groups have the same size (meaning the same proportion of the population is in each of the relapse risk groups). The relapse rate of each risk group is the median relapse rate of this group. Thus, for k relapse risk groups with relapse rates r_i the model equation for risk group i is given by:

$$\frac{d}{dt} S_i(t) = w(t; \mu, \sigma)/k - (r_i + n)\, S_i(t), \qquad S_i(0) = 0,$$

where $S_i(t)$ is the fraction of susceptibles who are in risk group i ($i \in \{1,2,\dots,k\}$) at time t, k is the number of relapse risk groups, r_i is the median relapse rate of group i, and n is the constant infection rate. This model equation describes that individuals are equally distributed to the risk groups, thus $w(t; \mu, \sigma)/k$ is the fraction of individuals who are susceptible and in risk group i after the lognormal distributed drug washout time. Individuals leave the compartment of susceptible individuals after a relapse (at rate r_i) or a new infection (at rate n). The overall fraction of susceptible individuals at time t, $S(t)$, is then given by the sum of all susceptible individuals in the different risk groups:

$$S(t) = \sum_{i=1}^{k} S_i(t).$$

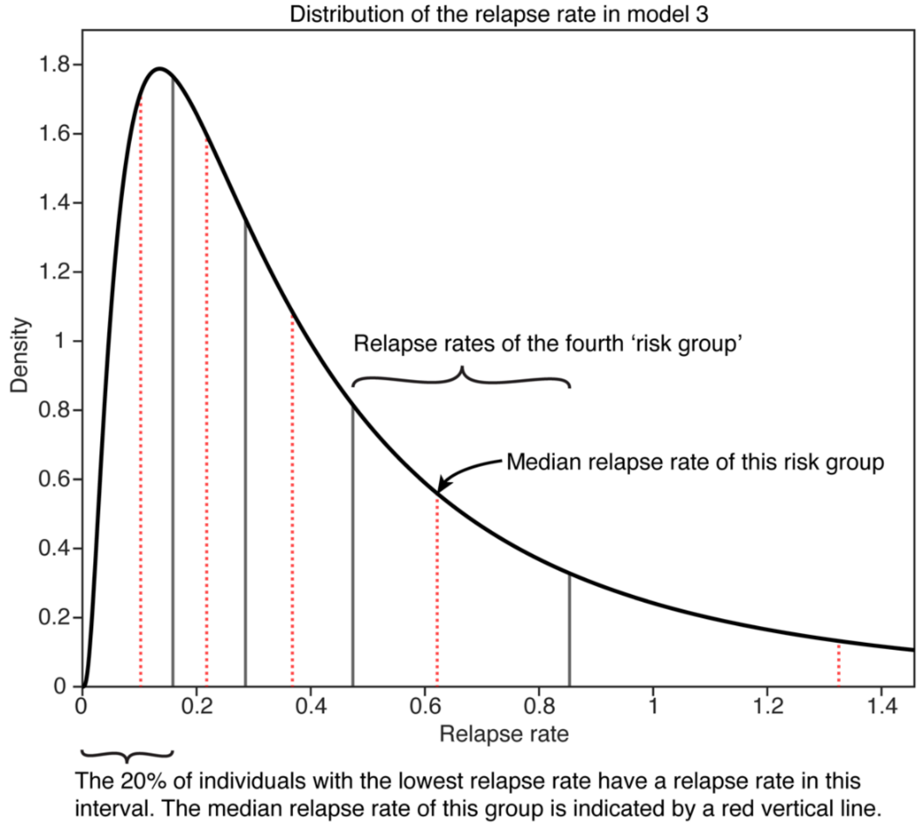

Fig B Scheme of the distribution of the relapse rates in model 3. Relapse rates in model 3 are lognormally distributed (black curve). The population is divided into 'relapse risk groups' of equal size (vertical grey lines). In this scheme there are five relapse risk groups. When numerically solving the model equation, individuals from the same relapse risk group are considered to have the same relapse rate which is chosen as the median relapse rate of that group (vertical red dotted lines).

Model 4: temporal and population heterogeneity

Model 4 takes both population heterogeneity and temporal heterogeneity in relapses into account as a combination and extension of models 2 and 3. As for model 3, we group the population in k different relapse risk groups of equal size. We use again percentiles of the relapse risk distribution (a lognormal distribution) to define the relapse risk groups. Individuals in the same relapse risk group have the same initial relapse risk that decreases over time as in model 2. Thus, the relapse risk of group i is given by:

$$r_i(t) = I_i e^{-dt},$$

where I_i is the initial relapse risk for relapse risk group i and d is the relapse risk decay rate that we assume to be the same for all individuals regardless of their initial relapse risk. Model 4 is given by:

$$\frac{d}{dt} S_i(t) = w(t; \mu, \sigma)/k - (r_i(t) + n)\, S_i(t), \qquad S_i(0) = 0,$$

where $S_i(t)$ is the fraction of susceptible individuals that are in risk group i ($i \in \{1,2, \dots, k\}$) at time t, k is the number of relapse risk groups, $r_i(t) = I_i e^{-dt}$ is the time-dependent relapse rate of group i, and n is the constant reinfection rate. As for model 3, the overall fraction of susceptible individuals at time t, $S(t)$, is the sum of all susceptible individuals in the different risk groups.

Models for two recurrences

To fit the models not only to the first recurrence after enrolment but to the first and second recurrence (only for the Thailand-Myanmar data because the Papua New Guinea data does not contain multiple recurrence times), we extend the models to take two recurrences into account. The model scheme is shown in **Fig C**.

For models 1 to 4, we extend the models with an additional compartment for protected and susceptible individuals (see **Fig C**). We make the following model assumptions:

- After the first recurrence, individuals are again protected. Thus, it is assumed that all recurrences are detected and immediately treated. We do not explicitly consider blood-stage infections and their duration.
- The drug washout time and time to recurrence follow the same distribution for the first and second recurrence. Thus, there is either no significant change in the hypnozoite number or the relapse rate is independent of the hypnozoite number. There is also no seasonality or changing of the infection rate over time.

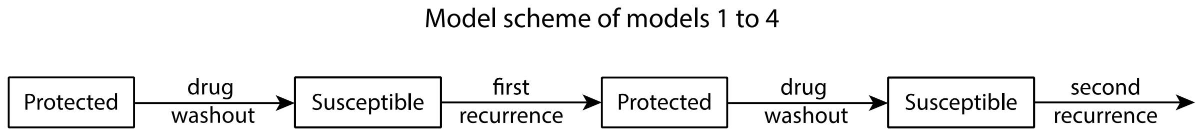

Fig C Model scheme of models 1 to 4 for two recurrences. After the first recurrence, we assume that all individuals are treated with the same drug as at enrolment. Both the drug washout rate and the recurrence rate for the second recurrence are the same as for the first recurrence for models 1 to 4.

Parameters of the models

The Thailand-Myanmar data consists of two different studies and individuals treated with different antimalarials. Different parameters of the models may vary by study or antimalarial treatment (see **Table A**).

For the constant infection rate, we considered both the case that the infection rate is the same for the two different studies and the case that is different (see **Table A** in **S4 Model comparisons**).

The drug washout time depends on the antimalarial treatment. In the case that individuals received a treatment with a combination of different drugs, the drug with the longer half-life determines the drug washout time. The half-lifes of the different antimalarials are 20-45 minutes for artesunate (AS) [1], 4-6 hours for primaquine (PMQ) [2, 3], approx. 3 weeks for dihydroartemisinin-piperaquine (DP) [4, 5], and 1-2 months for chloroquine (CHQ) [6]. Thus, for individuals treated with chloroquine and primaquine (CHQ/PMQ) the drug washout time is determined by chloroquine. For individuals treated with dihydroartemisinin-piperaquine and primaquine (DP/PMQ) the drug washout time is determined by dihydroartemisinin-piperaquine. Note that recurring *P vivax* infections were treated with the same antimalarial as at enrollment in the VHX study and with the standard chloroquine and primaquine in the BPD study. Thus, all individuals except the group treated DP/PMQ in the BPD study were treated with the same antimalarial at each recurrence. For simplicity, we fitted a separate drug washout time distribution for the DP/PMQ group, thus we assume that these individuals are treated with the same antimalarial at each treatment or at least an antimalarial with the same washout time distribution.

For the relapse parameters, we distinguish between individuals who received blood-stage treatment only (AS and CHQ) and those who also received primaquine (CHQ/PMQ and DP/PMQ). Since primaquine is a radical cure killing parasites of all stages including hypnozoites [7], individuals who were treated with primaquine are expected to not have any relapses.

Parameter dependency groups in the model fits to the Thailand-Myanmar data

Treatment	Study (group for new infection rate)	Drug washout distribution group	Relapse group
Artesunate	VHX	AS	Blood-stage treatment
Chloroquine	VHX	CHQ	Blood-stage treatment
Chloroquine & Primaquine	VHX	CHQ	-
Chloroquine & Primaquine	BPD	CHQ	-
Dihydroartemisinin-Piperaquine & Primaquine	BPD	DP	-

Table A This table shows how the parameters for new infections, the drug washout distribution, and relapses depend on treatment and study in the Thailand-Myanmar data. The parameters are the same if they are in the same group, e.g., all individuals treated with chloroquine have the same drug washout time distribution regardless of whether they were treated with chloroquine and primaquine or only with chloroquine.

The parameters for each model and each data set are given below.

Model 1: constant relapse rate

PNG data: Since in this model both the infection rate and the relapse rate are constant, we sum them to a constant recurrence rate (each individually would not be identifiable). The model contains two parameters for the drug washout time distribution, the recurrence rate for patients treated for blood-stage infection only, and the recurrence rate for patients treated for both blood- and liver-stage infection.

PNG data by village: The model contains two parameters for the drug washout time distribution for all villages and the recurrence rates for patients treated for blood-stage infection only and patients treated for both blood- and liver-stage infections for each of the 5 villages. Overall, model 1 fit to the PNG data by village contains 12 parameters.

Thailand-Myanmar data (for the case of two different infection rates): The model contains six parameters for the drug washout time, the mean and standard deviation for the lognormal distribution of drug washout times for AS, CHQ, and DP, respectively. Thus, we have the recurrence rate for individuals in the VHX study who received blood-stage treatment, the recurrence rate for the VHX study with primaquine treatment, and the recurrence rate for the BPD study with primaquine treatment. Overall, this model has 9 parameters.

Model 2: temporal heterogeneity

PNG data: The model contains two parameters for the drug washout time distribution, the rate of new infections, and for the time-dependent relapse rate $r(t) = Ie^{-dt}$, the initial relapse rate (I) and the rate of decay of the relapse rate (d) for blood-stage treatment. Overall, the model contains 5 parameters.

PNG data by village: The model contains two parameters for the drug washout time distribution for all villages and the rate of new infections, the initial relapse rate, and the exponential decay rate of the relapse rate for each of the 5 villages. Overall, model 2 fit to the PNG data by village contains 17 parameters.

Thailand-Myanmar data (for the case of two different infection rates): The model contains the mean and standard deviation for the lognormal distribution of drug washout times for AS, CHQ, and DP, respectively, and two infection rates for VHX and BPD. For the time-dependent relapse rate $r(t) = Ie^{-dt}$, we have the initial relapse rate (I) and the rate of decay of the relapse rate (d) for blood-stage treatment. Overall, there are 10 parameters in this model.

Model 3: population heterogeneity

PNG data: The model contains two parameters for the drug washout time distribution, the rate of new infections, and two parameters for the distribution of relapse rates.

PNG data by village: The model contains two parameters for the drug washout time distribution for all villages and the rate of new infections and two parameters for distribution of relapse rates for each of the 5 villages. Overall, model 3 fit to the PNG data by village contains 17 parameters.

Thailand-Myanmar data (for the case of two different infection rates): The model contains the mean and standard deviation for the lognormal distribution of drug washout times for AS, CHQ, and DP, respectively, and two infection rates for VHX and BPD. The relapse rate is lognormal distributed. Thus, the model also contains the mean and standard deviation for the relapse rate distribution for blood-stage. Overall, model 3 has 10 parameters.

This models also contains a parameter that is integer-valued, the number of relapse risk groups. We fit the model for different numbers of relapse risk groups and compared the model fits (see **Fig E** in **S4 Model comparisons**).

Model 4: temporal and population heterogeneity

PNG data: The model contains two parameters for the drug washout time distribution, the rate of new infections, two parameters for the distribution of the initial relapse rates, and the exponential decay rate of the relapse rate.

PNG data by village: The model contains two parameters for the drug washout time distribution for all villages and the rate of new infections, two parameters for distribution of relapse rates, and the decay rate of the relapse rate for each of the 5 villages. Overall, model 4 fit to the PNG data by village contains 22 parameters.

Thailand-Myanmar data (for the case of two different infection rates): The model contains the mean and standard deviation for the lognormal distribution of drug washout times for AS, CHQ, and DP, respectively, and two infection rates for VHX and BPD. The initial relapse rate is lognormal distributed and decays exponentially. Overall, model 4 has 11 parameters.

As model 3, this models also contains a parameter that is integer-valued, the number of relapse risk groups.

For a list of all the parameter values as well as their maximum likelihood estimates and their 95% confidence intervals, see **Tables A** to **D** in **S1 Tables** for the PNG data, **Tables E** to **H** in **S1 Tables** for the PNG data by village, and **Tables R** to **U** in **S1 Tables** for the Thailand-Myanmar data.

Likelihood function for fitting to the first and second recurrence time simultaneously (Thailand-Myanmar data only)

We use the following notation for the likelihood function:

- p: vector of parameters for the model
- D: data, D_i: data for individual i (there are overall N individuals who can be divided into N_0 individuals with no recurrences, N_1 individuals with 1 recurrence, and N_2 individuals with at least 2 recurrences)
- j: relapse risk group number out of different r risk groups of equal size (**Fig B**)
- R_i: relapse risk group of individual i
- $U_j(t)$: probability that an individual in risk group j has a recurrence more than t days after the previous recurrence (i.e., stays uninfected for at least t days).
  Note that for models 1 to 4 there is no difference between $U_j(t)$ for the first and the second recurrence as the drug washout time, relapses, and recurrences are assumed to have the save distribution for the first and the second recurrence.
- $G_j(t) = U_j(t-\Delta) - U_j(t)$: probability that an individual in risk group j has a recurrence between day $t-\Delta$ (the day of the last follow-up before a recurrence, Δ depends on the follow-up scheme) and day t (follow-up visit with a recurrence) after the previous recurrence.

The likelihood function is given by:

$$L(p|D) = P(D|p) = \prod_{i=1}^{N} P(D_i|p) = \prod_{i=1}^{N}\left[\sum_{j=1}^{r} P(D_i|R_i = j) \times P(R_i = j)\right],$$

where we assume that individuals are independent, we split up the population into the different 'relapse risk' groups R_i, and in the last step (and in the following) we omit the parameters p to keep the notation simpler.

For each individual, the data D_i contains the number of recurrences, recurrence times, and times of censoring. Thus, each individual will fall into one of the three groups below (omitting the index i for individual i in our notation for simplicity):

- 0 recurrences: denoted as $n = 0$, where n is the number of recurrences.
- 1 recurrence at time t_1: denoted as $(n = 1) \cap t_1$.
- At least 2 recurrences at times t_1 and t_2 (where t_1 is the time from the beginning of the study to the first recurrence and t_2 is the time from the first recurrence to the second recurrence): denoted as $(n \geq 2) \cap t_1 \cap t_2$

Next, we determine $P(D_i|R_i = j)$ for each of these three cases:

- 0 recurrences case:
  $$P(n = 0|\, R = j) = U_j(T),$$
  where T is the overall follow-up time.
- 1 recurrence case:
  $$\begin{aligned} P((n = 1) \cap t_1|\, R = j) &= P(n = 1 \mid t_1, R = j) \times P(t_1|R = j) \\ &= U_j(T - t_1) \times G_j(t_1). \end{aligned}$$
- At least 2 recurrences case:
  $$\begin{aligned} P((n \geq 2) \cap t_1 \cap t_2 \mid j) &= P(n \geq 2|\, t_1, t_2, j) \times P(t_1 \cap t_2|\, j) \\ &= G_j(t_1) \times G_j(t_2 - t_1), \end{aligned}$$

where in the last step $P(n \geq 2|\, t_1, t_2, j) = 1$ as the probability to have at least two recurrences given the time of two recurrences is 1. Alternatively, this can also be derived mathematically in the following way:

$$P(n \geq 2|\, t_1, t_2, R = j) = P(n = 2|\, t_1, t_2, R = j) + P(n > 2|\, t_1, t_2, R = j)$$
$$= U_j(T - (t_1 + t_2)) + \left[1 - U_j\big(T - (t_1 + t_2)\big)\right] = 1$$

as the probability to have exactly two recurrences is equal to the probability to have no more recurrences in the remaining time (from the second recurrence to censoring) and the probability to have more than two recurrences is the probability to have at least one recurrence in the remaining time (i.e., to not have no recurrences).

Now, we know $P(D_i|R_i = j)$ for each of the three cases. Splitting up the population into individuals with 0, 1, or at least 2 recurrences and using the above formulas for the likelihood function and $P(D_i|R_i = j)$, we obtain the following likelihood function:

$$L(p|D) = \prod_{i=1}^{N_0}\left[\sum_{j=1}^{r} U_j(T_i) \times P(R_i = j)\right] \times \prod_{i=1}^{N_1}\left[\sum_{j=1}^{r} G_j\big(t_{i,1}\big) \times U_j(T - t_1) \times P(R_i = j)\right]$$
$$\times \prod_{i=1}^{N_2}\left[\sum_{j=1}^{r} G_j(t_{i,1}) \times G_j(t_{i,2}) \times P(R_i = j)\right].$$

The general loglikelihood function is then:

$$l(p|D) = \sum_{i=1}^{N_0} \log\left[\sum_{j=1}^{r} U_j(T_i) \times P(R_i = j)\right]$$
$$+ \sum_{i=1}^{N_1} \log\left[\sum_{j=1}^{r} G_j\big(t_{i,1}\big) \times U_j(T_i - t_{i,1}) \times P(R_i = j)\right]$$
$$+ \sum_{i=1}^{N_2} \log\left[\sum_{j=1}^{r} G_j\big(t_{i,1}\big) \times G_j(t_{i,2}) \times P(R_i = j)\right].$$

In the case of only one risk group for the entire population, i.e., for models 1 and 2, the loglikelihood function simplifies to:

$$l(p|D) = \sum_{i=1}^{N_0} \log[U(T_i)] + \sum_{i=1}^{N_1} \log\left[U\big(t_{i,1}\big) \times U(T_i - t_{i,1})\right] + \sum_{i=1}^{N_2} \log\left[G\big(t_{i,1}\big) \times G(t_{i,2})\right].$$

In the case that individuals are equally distributed to the different risk groups (by using percentiles of the relapse rate distribution), i.e., in models 3 and 4, we have the following simplified loglikelihood function:

$$
\begin{aligned}
l(p|D) = &\sum_{i=1}^{N_0} \log\left[\sum_{j=1}^{r} U_j(T_i)\right] \\
&+ \sum_{i=1}^{N_1} \log\left[\sum_{j=1}^{r} G_j\big(t_{i,1}\big) \times U_j(T_i - t_{i,1})\right] \\
&+ \sum_{i=1}^{N_2} \log\left[\sum_{j=1}^{r} G_j\big(t_{i,1}\big) \times G_j(t_{i,2})\right] + (N_0 + N_1 + N_2) \times \log\left[\frac{1}{r}\right].
\end{aligned}
$$

The last term in the loglikelihood function for model 3 comes from $P(R_i = j)$ as it holds that

$$P(R_i = j) = \frac{1}{r}$$

for all individuals i and all risk groups j. In models 3 and 4, the risk groups were chosen by the percentiles of the relapse rate distribution, i.e., a fraction $1/r$ of the population is in each of the risk groups.

Model fits

In order to fit the models to the time-to-recurrence data for the first (and to the second for the Thailand-Myanmar data) recurrence, we numerically solve the model equations (see main text) using the ODE solver ode15s in Matlab (version R2018b) [8].
We obtain the fraction of susceptible individuals at time t, $S(t)$, as the numerical solution of the model equation. The fraction of individuals that remain uninfected at time t is then given as all the individuals who are still protected by the antimalarial treatment and all the susceptible individuals who have not yet been reinfected or had a relapse, i.e.,

$$U(t) = \left(1 - \int_0^t w(\tau; \mu, \sigma)\, d\tau\right) + S(t),$$

where $w(t; \mu, \sigma)$ is the probability density function of the lognormal distribution of drug washout times with parameters μ and σ.
We interpret $U(t)$ as the probability to be uninfected until time t and use it to define the probability to have an infection at the visit on day t ($G(t)$) as in the main text:

$$G(t) = U(t - \Delta) - U(t),$$

where $t - \Delta$ is the time of the last follow-up visit before day t.
Both $U(t)$ and $G(t)$ depend on the model parameters, $G(t)$ also depends on the follow-up scheme, and for the population heterogeneity model they both depend on the number of relapse risk groups. We tried either one rate of new infections or two different rates of new infections for the two different studies in the Thailand-Myanmar data, different follow-up schemes and different numbers of relapse risk groups in the population heterogeneity model (see Supplementary results). In the end, we chose a daily follow-up scheme, 10 different relapse risk groups, two rates of new infections in the Thailand-Myanmar data, and one rate of new infections for the PNG data for all model comparisons and all data sets.
With $U(t)$ and $G(t)$ we can use the above loglikelihood function to fit our models to the first and second recurrence time in the Thailand-Myanmar data and obtain Maximum Likelihood Estimates (MLEs) for the parameter values. We do so by selecting random initial parameter values and minimizing the negative loglikelihood function using the Matlab function fmincon. In order to assure that we obtain a good fit, we minimize the negative loglikelihood function for 100 random initial parameter vectors and the MLE of the parameters for the fit to the first recurrence only (see **Tables N**, **O**, **P** and **Q** in **S1 Tables**).
We fit to the first recurrence times in the same way as to the first and second recurrence time in the Thailand-Myanmar data. However, instead of the above loglikelihood function, we used the following simpler loglikelihood function:

$$l(p|D) = \sum_{i=1}^{N_1} \log[U(t_i - \Delta) - U(t_i)] + \sum_{i=1}^{N_0} \log\,[U(T_i)],$$

where N_0 and N_1 are the numbers of individuals with zero and at least one recurrence, respectively, $U(t)$ is the probability to be uninfected until time t, t_i is the time of the first recurrence of individual i, $t_i - \Delta$ is the time of the last follow-up visit before day t_i, and T_i is the follow-up time of individual i. As for the model comparison for the fit to the first and second recurrence time, we use daily follow-up and 10 relapse risk groups in the population heterogeneity model.
We compare the model fits using the Akaike Information Criterion (AIC) that is given by

$$AIC = 2 \times \left(-l(p|D)\right) + 2 \times n_{par},$$

where $-l(p|D)$ denotes the negative loglikelihood and n_{par} denotes the number of parameters.

We also compared model fits of model 4 with model 3 using the likelihood-ratio test and found that model 4 is a significantly better model (p-value < 0.0001).

Confidence Intervals

We computed confidence intervals using bootstrapping and the percentile method. We drew individuals from the data with replacement and fitted each model to the new data as described above. However, we used only 10 random initial parameter values and the best fitting parameter values for the original data for fitting each model to the bootstrapped data (for efficiency and time reasons). This was repeated 1000 times. The 95% confidence interval for each parameter is the 95th percentile of the MLE of the best fitting parameters for the bootstrapped data.

Model simulations

We simulated 1,000 cohorts of 1,000 individuals for 1 year and artesunate or chloroquine using models 1 to 3. The parameters for the model simulation are the MLEs of the parameters from the fit to the first and second recurrence time in the Thailand-Myanmar data (see **Tables R**, **S** and **T** in **S1 Tables**). The detailed description of the model simulation method for each model is given below.

Model 1: constant relapse rate

For each individual a drug washout time is drawn from a lognormal distribution with the appropriate parameters depending on the antimalarial treatment. Since the recurrence rate is constant, the time from drug washout to a recurrence is exponentially distributed. Thus, the recurrence time is drawn from an exponential distribution with mean 1/recurrence rate. The time of the first recurrence is then the sum of the drug washout time and the time from drug washout to recurrence. This process is repeated until the individual has been simulated for 1 year.

Model 2: temporal heterogeneity

The drug washout time is drawn from a lognormal distribution as for model 1. In this model, the rate of new infections is constant, but the relapse rate is non-constant and decreases in time. For an event occurring at rate $r(t)$ the cumulative distribution function of the time to next event distribution is given by:

$$f(t) = 1 - e^{-\int_0^t r(x)dx}.$$

The recurrence rate is the sum of the rate of new infections and the relapse rate, thus the cumulative distribution function of the time to the next recurrence after drug washout is given by:

$$f(t) = 1 - e^{-\int_0^t n + Ie^{-dx}\, dx}.$$

Due to the prophylactic effect of the antimalarial treatment, there are no recurrences before drug washout. Thus, we shift the recurrence rate by the drug washout time w and to take into account the blocking of relapses and new infections before drug washout. We obtain the following cumulative distribution function for the time to the next recurrence:

$$g(t, w) = 1 - e^{-\int_0^t n + Ie^{-d(x+w)}\, dx} = 1 - e^{-nt - \frac{I}{d}e^{-dw}\left(1 - e^{-dt}\right)},$$

where w is drug washout time.

Next, we simulate the time to the next recurrence using inverse transform sampling, i.e., we sample a number x from a uniform distribution between 0 and 1 and estimate $g^{-1}(x, w)$ which is a sample of a random variable with cumulative distribution function $g(\cdot, w)$. We estimate $g^{-1}(x, w)$ by computing $g(\cdot, w)$ for a range of time points and choosing the time point t for which $g(t, w)$ is closest to x. Thus, the time to the first recurrence is given by the estimate for $g^{-1}(x, w)$. As for model 1, this process is repeated until the individual has been simulated for 1 year.

Model 3: population heterogeneity

Each individual has a different relapse rate that is sampled from the lognormal distribution of relapse rates. The relapse rate is constant and each individual keeps the same relapse rate for

the entire simulated year. The drug washout time was computed as for models 1 and 2. Since both the rate of new infections and relapse rate are constant, the time to the next recurrence can be simulated as for model 1 by sampling from an exponential distribution.

Instead of simulating the time to the next recurrence, we can also simulate the time to the next new infection and the time to the next relapse in the same way as described above. The time to the next recurrence is then the smaller of these. This approach has the advantage that we know for each simulated recurrence whether it is a relapse or a new infection.
For model 1, we interpret the recurrence rate of primaquine treated patients as the rate of new infections. The rate of relapses is then the recurrence rate for artesunate or chloroquine treated patients minus the rate of new infections.
The time to relapse in model 2 can be simulated exactly as described above and the time to a new infection is a sample from an exponential distribution as the rate of new infections is constant.

References

1. Morris CA, Duparc S, Borghini-Fuhrer I, Jung D, Shin C-S, Fleckenstein L. Review of the clinical pharmacokinetics of artesunate and its active metabolite dihydroartemisinin following intravenous, intramuscular, oral or rectal administration. Malaria Journal. 2011;10:263. doi: 10.1186/1475-2875-10-263.
2. Fletcher KA, Price Evans DA, Gilles HM, Greaves J, Bunnag D, Harinasuta T. Studies on the pharmacokinetics of primaquine. Bull WHO. 1981;59(3):407-12. PubMed Central PMCID: PMCPMC2396059.
3. Mihaly GW, Ward SA, Edwards G, Nicholl DD, Orme ML, Breckenridge AM. Pharmacokinetics of primaquine in man. I. Studies of the absolute bioavailability and effects of dose size. Br J Clin Pharmacol. 1985;19(6):745-50. doi: 10.1111/j.1365-2125.1985.tb02709.x.
4. Zani B, Gathu M, Donegan S, Olliaro PL, Sinclair D. Dihydroartemisinin-piperaquine for treating uncomplicated *Plasmodium falciparum* malaria. Cochrane Database of Systematic Reviews. 2014;(1). doi: 10.1002/14651858.CD010927.
5. Davis TME, Hung T-Y, Sim I-K, Karunajeewa HA, Ilett KF. Piperaquine: A Resurgent Antimalarial Drug. Drugs. 2005;65(1):75-87. doi: 10.2165/00003495-200565010-00004.
6. White NJ. Antimalarial pharmacokinetics and treatment regimens. Br J Clin Pharmacol. 1992;34(1):1-10. doi: 10.1111/j.1365-2125.1992.tb04100.x.
7. Price RN, Commons RJ, Battle KE, Thriemer K, Mendis K. *Plasmodium vivax* in the Era of the Shrinking *P. falciparum* Map. Trends in Parasitol. 2020;36(6):560-70. doi: 10.1016/j.pt.2020.03.009.
8. MATLAB R2018b (version 9.5.0.944444). Natick, Massachusetts, United States: The MathWorks, Inc.; 2018.
