# Supplementary material for: Population heterogeneity in Plasmodium vivax relapse risk
Source: PLoS Negl Trop Dis. 2022 Dec 19;16(12):e0010990. doi: 10.1371/journal.pntd.0010990 (PMC9810152; doi:10.1371/journal.pntd.0010990)
Supplement: S1 Model comparisons — (PDF) [file pntd.0010990.s004.pdf]

S4 Model comparisons

Different infection rate for the different studies or one infection rate for both studies in the Thailand-Myanmar data

We fit the models to the data both using one infection rate (for new, mosquito-borne infections) as well as two different infection rates for the two different studies in the Thailand-Myanmar data (as Taylor et al. [1]). With two infection rates each model contains one more parameter and the AIC decreases by 5, 7, and 2 for models 1, 2, and 3, respectively (see **Table A**). The model fits to first and second recurrence show very little difference for the fit with 1 or 2 infection rates (see **Fig A**). For this reason and due to the lower AICs, we do all model fits with two infection rates.

Comparison of the model fit with 1 or 2 infection rates (Thailand-Myanmar data)

Model	AIC with 1 infection rate	AIC with 2 infection rates
1: constant relapse rate	8799	8794
2: temporal heterogeneity	8499	8492
3: population heterogeneity	8428	8426

Table A Comparison of the model fit with 1 or 2 infection rates by AIC for the Thailand-Myanmar data. Including a second infection rate is one additional parameter for each model and improves the AIC by 5, 7, and 2 for models 1, 2, and 3, respectively. These model fits were done with daily follow-up and 10 relapse risk groups in model 3.

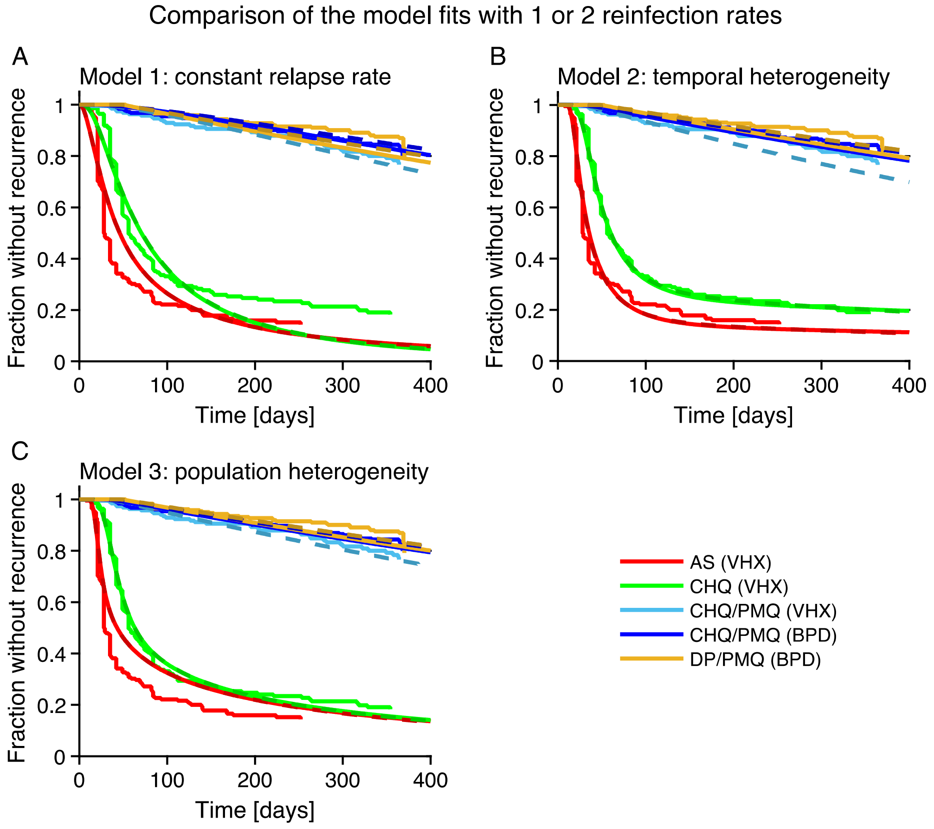

Fig A Comparison of the model fits with 1 infection rate for both studies and different infection rates for the two studies in the Thailand-Myanmar data. The data shown here are the time to the first recurrence survival curves for different antimalarial treatments. The model fit for one infection rate is shown in the same color as the corresponding data. Note that for one infection rate there is no difference between individuals treated with chloroquine and primaquine in the VHX study and the BPD study. The model fit for two different infection rates is shown as a dashed and darker line.

Follow-up schemes in the Thailand-Myanmar data

To compute the loglikelihood function and fit the models to the data, we need the follow-up scheme. According to Chu et al. [2], in the BPD study follow-up visits took place in weeks 2 and 4, then every four weeks. However, the data for the BPD study do not fit this follow-up scheme as recurrences were found also in weeks without follow-up visits. For the VHX study, it is not clear which follow-up scheme was used (the data also do not fit the follow-up scheme described for the BPD study). For this reason, we considered seven different follow-up schemes, fit the models using each of these follow-up schemes, and compare the model fits.
We considered the following follow-up schemes:

1. Daily follow-up
2. Weekly follow-up
3. Fortnightly follow-up
4. 4-weekly follow-up
5. Follow-up at the beginning of weeks 2 and 4, then every 4 weeks
6. Follow-up in the middle of weeks 2 and 4, then every 4 weeks
7. Follow-up at the end of weeks 2 and 4, then every 4 weeks

For each of these follow-up schemes, we fit the models to the data as described above. The different follow-up schemes affect the AIC such that is not possible to compare the fit of the models with different follow-up schemes based on the AIC as follow-up schemes with a longer time between visits result in a lower AIC (see **Table B**).

Comparison of the different follow-up schemes by AIC (Thailand-Myanmar data)

**Follow-up scheme**	**Model 1:** constant relapse rate	**Model 2:** temporal heterogeneity	**Model 3:** population heterogeneity
1	8794	8492	8426
2	5865	5610	5544
3	4787	4596	4532
4	3712	3653	3606
5	4289	4040	3990
6	4602	4319	4264
7	5023	4721	4663

Table B Comparison of the different follow-up schemes by AIC (Thailand-Myanmar data). The AICs are affected by the follow-up scheme such that the model cannot be compared using the AIC. We used different infection rates for the two studies for these model fits and 10 relapse risk groups for model 3.

The model fits for models 1 to 3 with the different follow-up schemes are shown in **Figs B**, **C** and **D**, respectively. There is little qualitative difference between the model fits with the different follow-up schemes. For each of the seven proposed follow-up schemes, the conclusion that model 3 gives the best fit with the lowest AIC and that only models 2 and 3 show a biphasic decay in the survival curves with a steeper initial decay and then a slower decay holds. Hence, we choose the first follow-up scheme (daily follow-up) and use this follow-up scheme for all model comparisons and model fits.

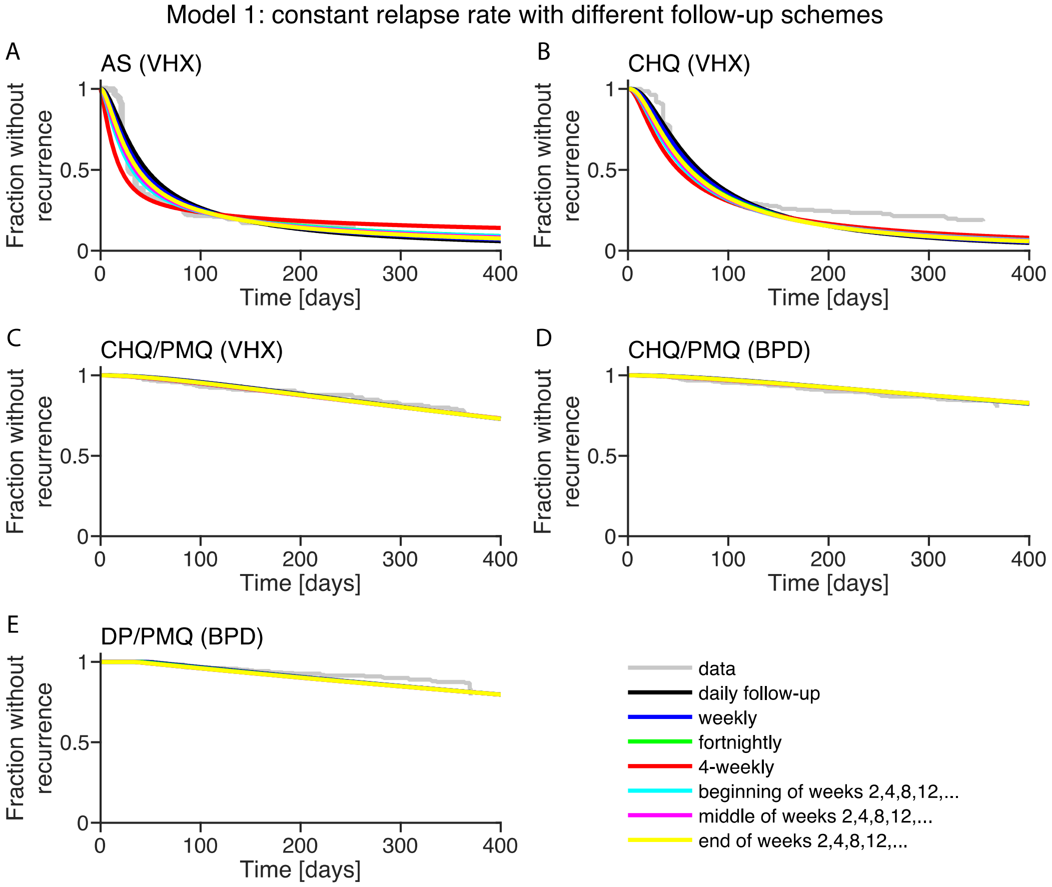

Fig B Fit of model 1 using different follow-up schemes for each antimalarial treatment and study in the Thailand-Myanmar data. The first recurrence data is shown in gray for comparison.

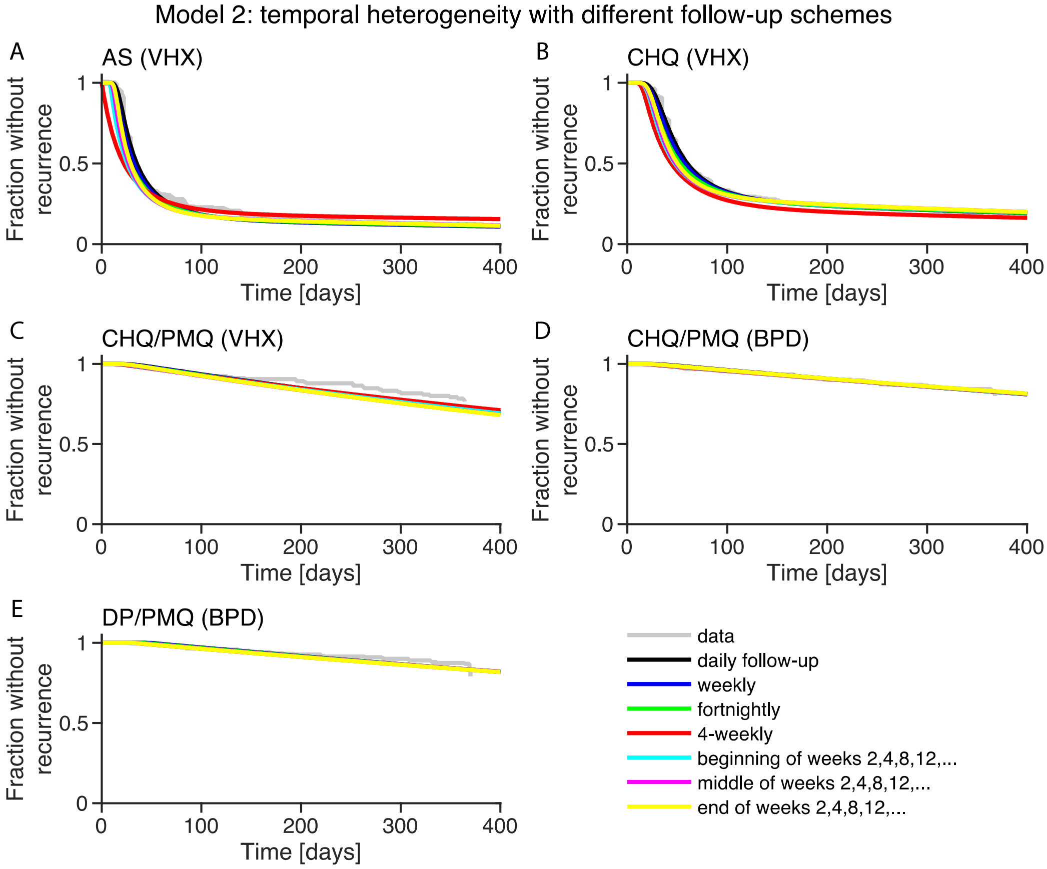

Fig C Fit of model 2 using different follow-up schemes for each antimalarial treatment and study in the Thailand-Myanmar data. The first recurrence data is shown in gray for comparison.

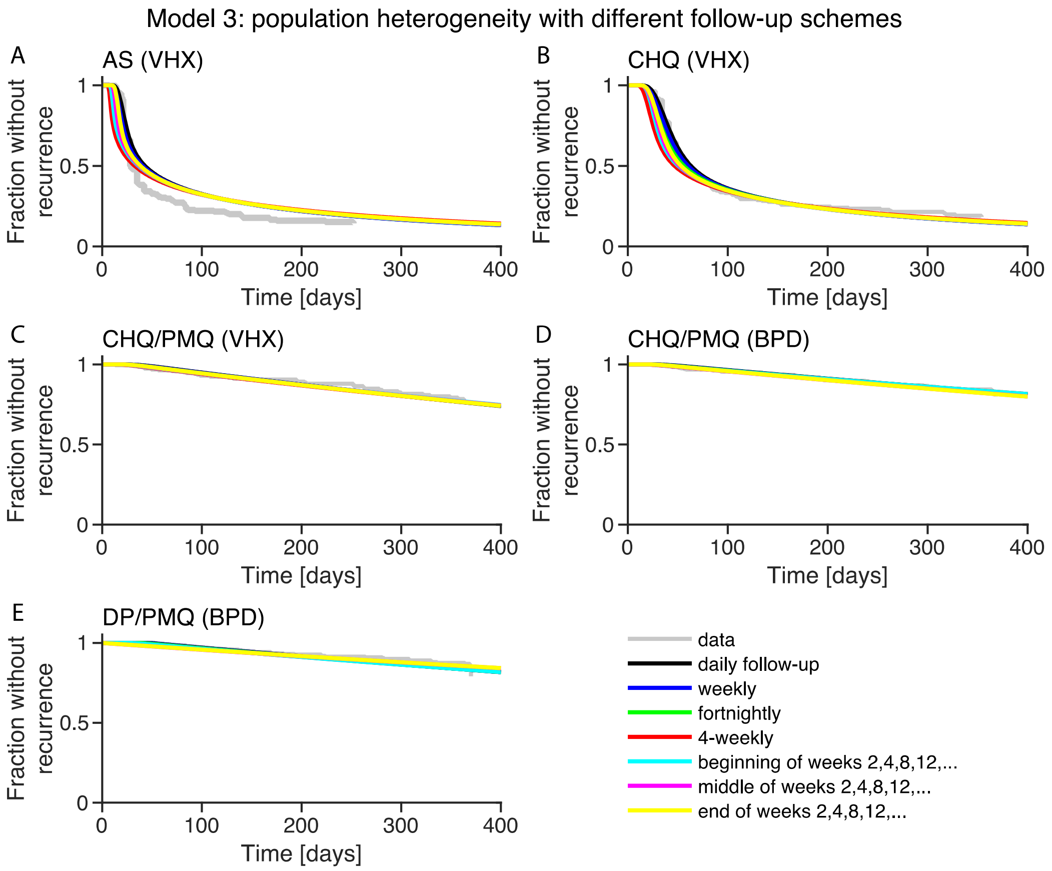

Fig D Fit of model 3 using different follow-up schemes for each antimalarial treatment and study in the Thailand-Myanmar data. The first recurrence data is shown in gray for comparison.

Different numbers of relapse risk groups in the population heterogeneity model for the Thailand-Myanmar data

In model 3, the relapse rate is constant but drawn from a lognormal distribution to model population heterogeneity. Individuals are grouped into k relapse risk groups of equal size (see **Fig B** in **S3 Methods**). We fit model 3 for $k = 5, 10, 15,$ and 20 groups. Since the AICs are the same and the model fits are very similar (see **Table C** and **Fig E**), we use $k = 10$ for all model fits, comparisons, and simulations.

AICs for model 3 with different numbers of relapse risk groups (Thailand-Myanmar data)

Numbers of relapse risk groups	AIC
5	8426
10	8426
15	8426
20	8426

Table C Comparison of the AICs for model 3 with different numbers of relapse risk groups in the Thailand-Myanmar data. Model 3 was fit with two infection rates and daily follow-ups.

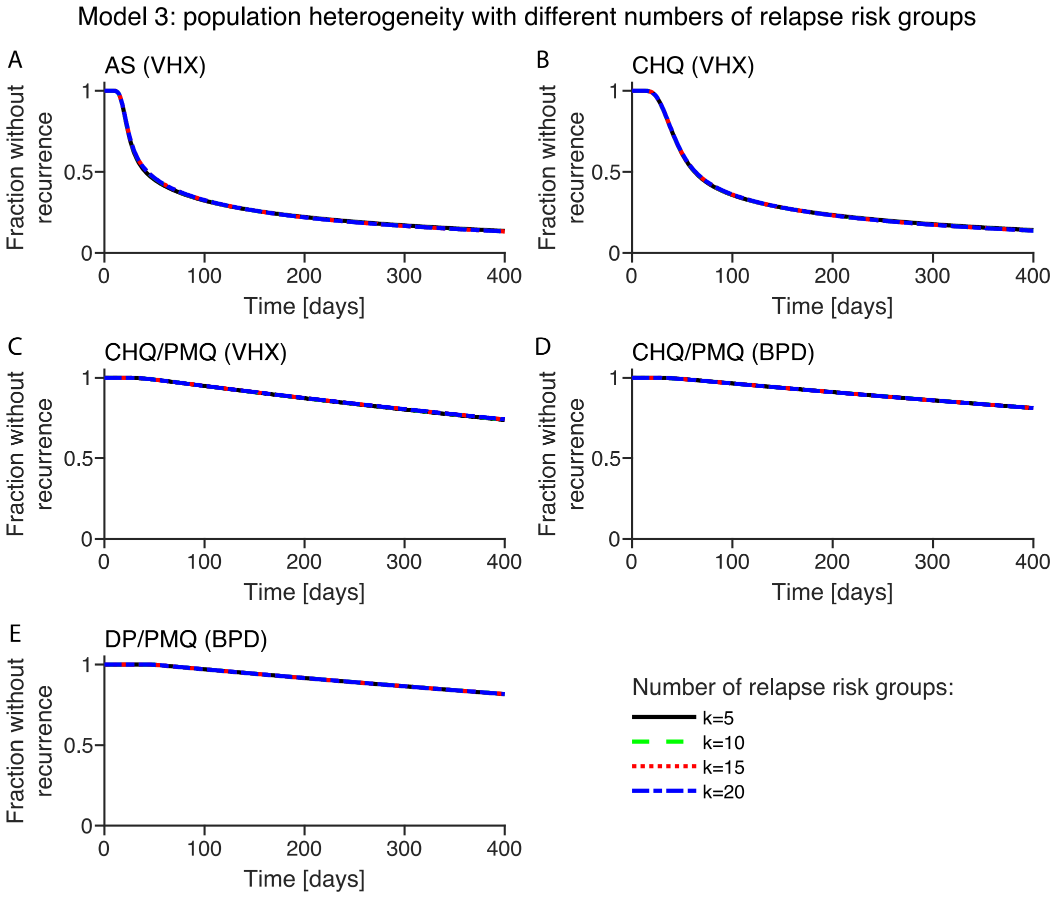

Fig E Comparison of the model fit of model 3 with different numbers of relapse risk groups (Thailand-Myanmar data). Each subfigure contains the best model fit of model 3 to one treatment group for different numbers of relapse risk groups, $k = 5, 10, 15,$ and 20. For these model fits, we used different infection rates for the different studies and daily follow-up.

Comparison of the population heterogeneity model fit to the Thailand-Myanmar data with lognormal, gamma, and exponential distribution for the relapse rates

For the model comparisons, we used a lognormal distribution for relapse rates in the population heterogeneity model. We also fitted the population heterogeneity model using the gamma and exponential distributions as the distribution of relapse rates. The lognormal and the gamma distributions of relapse rates give a similar fit to the Thailand-Myanmar data, with the lognormal distributed relapse rates model having a slightly lower AIC than the model with gamma distributed relapse rates (see **Fig F**). Furthermore, both models have the same number of parameters and similar parameter value estimates (see **Table D**). The exponential distribution has one less parameter but does not give as good a fit as either the lognormal or the gamma distribution.

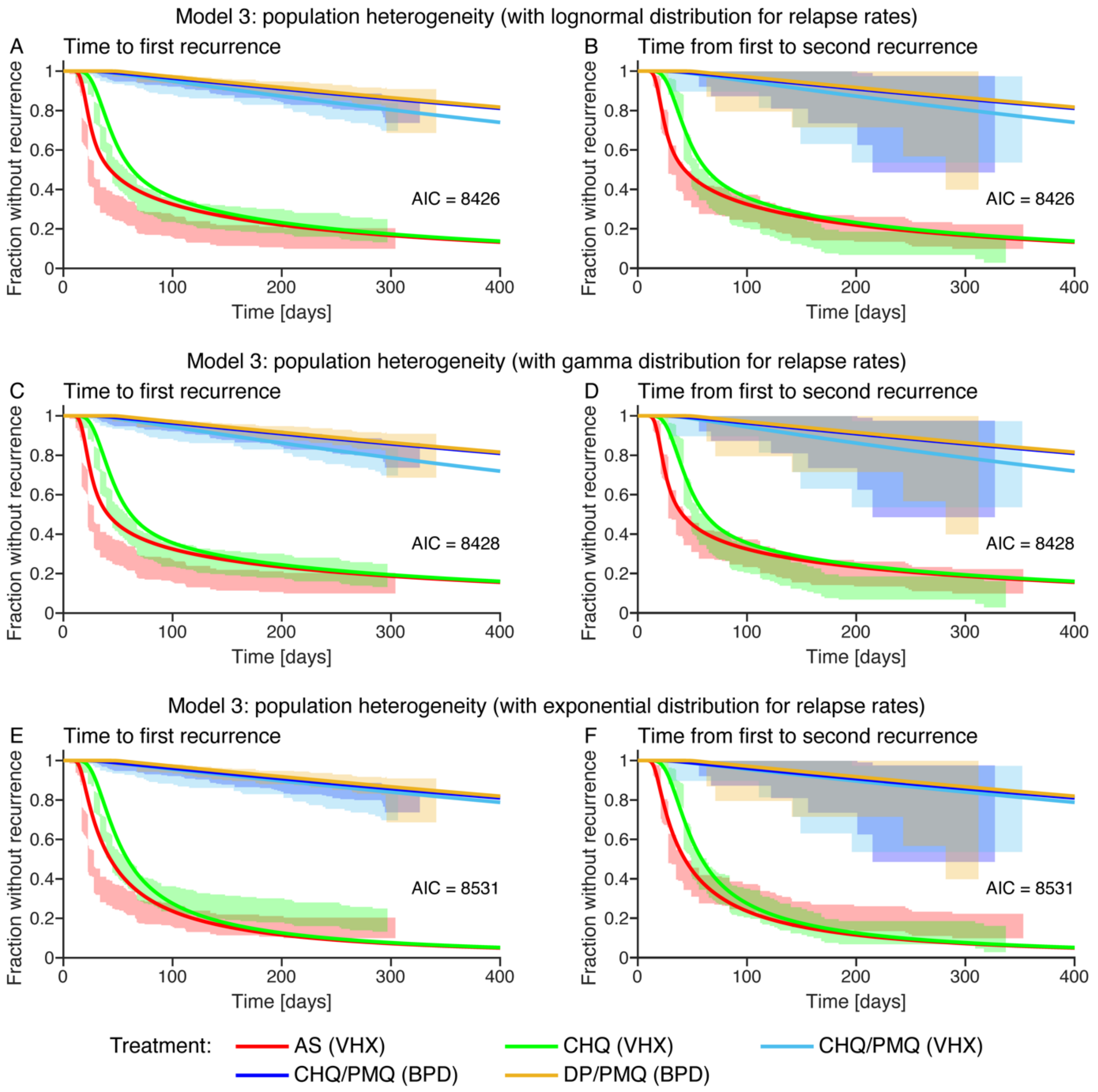

Fig F Fit of the population heterogeneity model with a lognormal, a gamma, and an exponential distribution of the relapse rates to the first and second recurrence time in the Thailand-Myanmar data. For these model fits, we used different infection rates for the different studies, daily follow-up, and 10 relapse risk groups.

Parameters of model 3: population heterogeneity (Thailand-Myanmar data, lognormal, gamma, and exponential distribution of relapse rates)

Parameter	Lognormal d.	Gamma distr.	Exp. distr.
Mean of the logarithmic values of the drug washout time distribution for AS	2.95	2.90	2.72
Standard deviation of the logarithmic values of the drug washout time distribution for AS	0.24	0.24	0.22
Mean of the logarithmic values of the drug washout time distribution for CHQ	3.53	3.51	3.34
Standard deviation of the logarithmic values of the drug washout time distribution for CHQ	0.33	0.34	0.31
Mean of the logarithmic values of the drug washout time distribution for DP	3.88	3.88	3.89
Standard deviation of the logarithmic values of the drug washout time distribution for DP	0.060	0.059	0.061
Rate of new infections in the VHX study [per day]	0.0008	0.0009	0.0006
Rate of new infections in the BPD study [per day]	0.0006	0.00058	0.0006
Relapse rate distribution for AS and CHQ, parameter 1	-3.73	0.30	0.035
Relapse rate distribution for AS and CHQ, parameter 2	2.80	0.38	-

Table D Parameter estimates for the parameters of the population heterogeneity model fit simultaneously to the first and second recurrence time in the Thailand-Myanmar data for a lognormal distribution of relapse rates and a gamma distribution of relapse rates. The parameter estimates are the maximum likelihood estimates for the model fits with different infection rates for the different studies, daily follow-up, and 10 relapse risk groups. Abbreviations: AS artesunate treatment, CHQ chloroquine treatment, DP dihydroartemisinin-piperaquine treatment, PMQ primaquine treatment, VHX Vivax History study, BPD Best Primaquine Dose study.

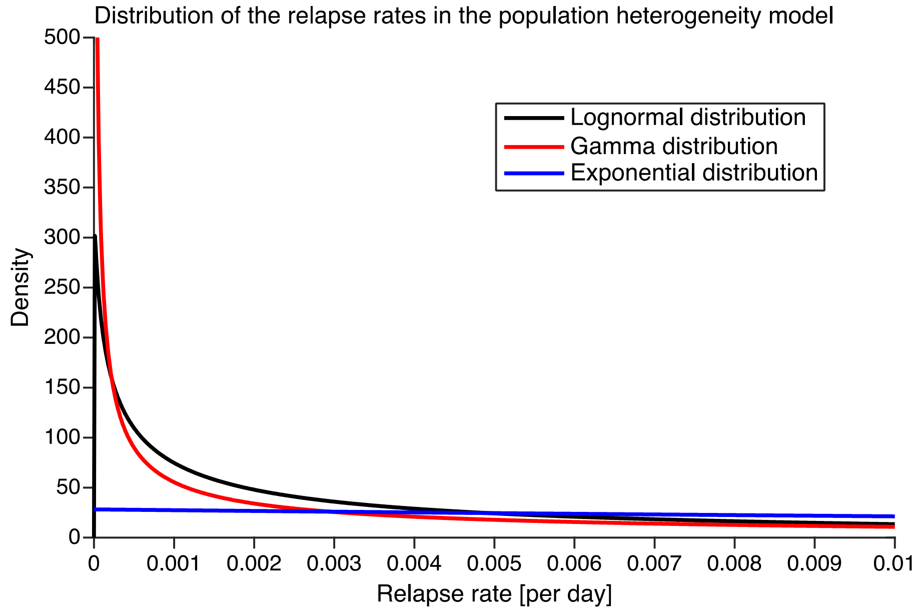

Fig G Comparison of the relapse rate distribution in the population heterogeneity model with a lognormal, a gamma, and an exponential distribution of relapse rates.

References

1. Taylor AR, Watson JA, Chu CS, Puaprasert K, Duanguppama J, Day NPJ, et al. Resolving the cause of recurrent *Plasmodium vivax* malaria probabilistically. Nature Communications. 2019;10(1):5595. doi: 10.1038/s41467-019-13412-x.

2. Chu CS, Phyo AP, Turner C, Win HH, Poe NP, Yotyingaphiram W, et al. Chloroquine Versus Dihydroartemisinin-Piperaquine With Standard High-dose Primaquine Given Either for 7 Days or 14 Days in *Plasmodium vivax* Malaria. Clinical Infectious Diseases. 2019;68(8):1311-9. doi: 10.1093/cid/ciy735.
